# Refined saddle-point preconditioners for discretized Stokes problems

**DOI:** 10.1007/s00211-017-0908-4

**Published:** 2017-07-25

**Authors:** John W. Pearson, Jennifer Pestana, David J. Silvester

**Affiliations:** 1 School of Mathematics, Statistics and Actuarial Science, University of Kent, Sibson Building, Parkwood Road, Canterbury, CT2 7FS UK; 2 Department of Mathematics and Statistics, University of Strathclyde, Glasgow, G1 1XH UK; 3 School of Mathematics, University of Manchester, Oxford Road, Manchester, M13 9PL UK

**Keywords:** 65F10, 65F15, 65N30

## Abstract

This paper is concerned with the implementation of efficient solution algorithms for elliptic problems with constraints. We establish theory which shows that including a simple scaling within well-established block diagonal preconditioners for Stokes problems can result in significantly faster convergence when applying the preconditioned MINRES method. The codes used in the numerical studies are available online.

## Introduction

The motivation for this work is the development of fast and robust linear solvers for stabilized mixed approximations of the Stokes equations,

$$\begin{aligned} -\nabla ^{2}\vec {v}+\nabla {}p={}&\vec {f}, \\ -\nabla \cdot \vec {v}={}&0, \end{aligned}$$

together with suitable (Dirichlet, Neumann or mixed) boundary conditions. Stokes problems typically arise when modelling the flow of a slow-moving fluid such as magma in the Earth’s mantle, see [18]. In our setting $\vec {v}$ denotes the flow velocity, *p* is the pressure, and $\vec {f}$ represents a source term that drives the PDE system. The associated boundary value problem is usually posed on a bounded domain $\Omega \subset {\mathbb {R}}^{\bar{d}}$, $\bar{d}\in \{2,3\}$. Stokes problems also arise in a natural way when the (unsteady) Navier–Stokes equations are simplified using classical operator splitting techniques, see [6].

We suppose that the boundary value problem is discretized using standard mixed finite elements. That is we take $\{\phi _{i}\}_{i=1,...,n_v}$ as the finite element basis functions for the velocity components (we assume that the same approximation space is used for each one), and $\{\psi _{i}\}_{i=1,...,m}$ for the pressure; so that $n_v$ and *m* are the number of velocity and pressure grid nodes respectively. Having set up the associated velocity basis set (for example, $\{ \vec { \phi }_1, \ldots , \vec { \phi }_{2 n_v}\} := \{ ({\phi }_1, 0)^T, \ldots , ({\phi }_{n_v}, 0)^T, (0, {\phi }_{1})^T, \ldots , (0, {\phi }_{n_v})^T\}$ in two dimensions), the resulting discrete Stokes system is the *saddle-point* system,

1.1 $$\begin{aligned} \begin{bmatrix} A&\quad B^T\\ B&\quad -C \end{bmatrix} \begin{bmatrix} {\varvec{v}}\\ {\varvec{p}}\end{bmatrix}= \begin{bmatrix} {\varvec{f}}\\ {\varvec{g}} \end{bmatrix}, \end{aligned}$$

where $A\in {\mathbb {R}}^{n\times {}n}$ (with $n=\bar{d}n_v$) is the *vector-Laplacian matrix* given by

$$\begin{aligned} A=[a_{ij}],\quad \quad a_{ij}=\int _{\Omega }\nabla \vec { \phi _i} : \nabla \vec { \phi _j}~\mathrm{d}\Omega , \end{aligned}$$

and $B\in {\mathbb {R}}^{m\times {}n}$ is the *divergence matrix*

$$\begin{aligned} B=[b_{ij}],\quad \quad b_{ij}=-\int _{\Omega }\psi _i\nabla \cdot \vec { \phi _j}~\mathrm{d}\Omega . \end{aligned}$$

The vectors ${\varvec{v}}$, ${\varvec{p}}$ are discretized representations of $\vec {v}$, *p*, with ${\varvec{f}}$, ${\varvec{g}}$ taking into account the source term $\vec {f}$ as well as nonhomogeneous boundary conditions. The matrix *C* is the zero matrix when a stable finite element discretization (such as the $Q_2 \textendash Q_1$ Taylor–Hood element) is used, and is the *stabilization matrix* otherwise. We assume that *A* is symmetric positive definite, which is the case when a Dirichlet condition is imposed on at least part of the boundary. The matrix *C* is always positive semi-definite. For consistency with the continuous Stokes system the matrix *B* should satisfy ${\varvec{1}}\in {{\mathrm{null}}}(B^T)$ in the case of enclosed flow (see, e.g., [8, Chapter 3]). However, other vectors may also lie in the nullspace of *B*; these are artefacts of the discretization, or arise from the imposition of essential boundary conditions.

The matrix system (1.1) is of classical *saddle-point form*.^1^ There has been a great deal of research devoted to solving systems of the form (1.1) using preconditioned iterative methods; see [2] for a definitive review. This body of work is relevant to any linear system that is generated by a mixed approximation; see [4, Chapter 3] for a characterization. To state the key spectral properties, it is useful to let

$$\begin{aligned} {{\mathcal {A}}}= \begin{bmatrix} A&\quad B^T\\ B&\quad -C \end{bmatrix}, \end{aligned}$$

where $A\in {\mathbb {R}}^{n\times n}$ is symmetric positive definite as above, $C\in {\mathbb {R}}^{m\times m}$ is symmetric positive semidefinite, $B\in {\mathbb {R}}^{m\times n}$ with $m \le n$ and $\text {rank}(B) = r\le m$. We suppose that the (negative) Schur complement of ${{\mathcal {A}}}$,

1.2 $$\begin{aligned} S=BA^{-1}B^{T}+C, \end{aligned}$$

has rank $p$. Then under these conditions ${{\mathcal {A}}}$ has *n* positive eigenvalues, $p$ negative eigenvalues and $m-p$ zero eigenvalues [2, page 21].

A widely studied block diagonal preconditioner for ${{\mathcal {A}}}$ is given by

1.3 $$\begin{aligned} {\mathcal {P}}_1 = \begin{bmatrix}A&\quad 0 \\ 0&\quad H \end{bmatrix}, \end{aligned}$$

where $H\in {\mathbb {R}}^{n\times {}n}$ is some symmetric positive definite approximation to the Schur complement *S*. In the case where $H=S$ and $C=0$ (whereby *B* must be full rank for *S*, and hence ${\mathcal {P}}_1$, to be invertible), it is known that the eigenvalues of the preconditioned system are given by [14, 17]

1.4 $$\begin{aligned} \lambda ({\mathcal {P}}_1^{-1}{{\mathcal {A}}})\in \left\{ 1,\frac{1}{2}(1\pm \sqrt{5})\right\} , \end{aligned}$$

and in the case where the approximation of *S* (or indeed *A*) is inexact the preconditioner is frequently found to be extremely effective also. When the condition on *C* is weakened to allow the matrix to be symmetric positive semi-definite, it can be shown that^2^

1.5 $$\begin{aligned} \lambda ({\mathcal {P}}_1^{-1}{{\mathcal {A}}})\in \left[ -1,\frac{1}{2}(1-\sqrt{5})\right] \cup \left[ 1,\frac{1}{2}(1+\sqrt{5})\right] . \end{aligned}$$

We note that, for the results (1.4) and (1.5), we have assumed invertibility of *S* in order for ${\mathcal {P}}_1$ itself to be invertible, as ${\mathcal {P}}_1$ includes the exact Schur complement. In the remainder of this paper, however, we consider situations where the Schur complement could be singular, but construct an inexact approximation *H* which is invertible.

In the specific case of the Stokes equations, the approximate Schur complement is either the *mass matrix* associated with the pressure approximation space^3^

$$\begin{aligned} \ Q=[m_{p,ij}],\quad \quad {}m_{p,ij}=\int _{\Omega }\psi _i\psi _j~\mathrm{d}\Omega , \end{aligned}$$

or an approximation. Common approximations of *Q* are its diagonal (see [23, 27]), a lumped version (see [24]), or a Chebyshev semi-iteration method applied to *Q* (see [12, 13, 30]). We will study a refined version of the classical preconditioner in this work: instead of taking $S\approx {}H$, our idea is to incorporate a scaling constant $\alpha >0$ and investigate using

1.6 $$\begin{aligned} {{\mathcal {P}}}_\alpha = \begin{bmatrix}A&\quad 0 \\ 0&\quad \alpha H\end{bmatrix} \end{aligned}$$

as a potential preconditioner for ${{\mathcal {A}}}$. Intuitively there is little reason to assume that the matrix ${{\mathcal {P}}}_\alpha$ would be a more effective preconditioner than ${\mathcal {P}}_1$: by scaling the Schur complement we are after all moving the preconditioner ‘further’ from the *ideal* preconditioner ${\mathcal {P}}_1$. Remarkably, however, we frequently observe a significant improvement in the Stokes case. This improvement is justified theoretically herein. We also explain why setting a large value of $\alpha$ can significantly improve the performance of the iterative solver when a stabilized mixed approximation is employed.

We highlight a related discussion on [23, page 1361] where a small scaling parameter was considered: the motivation for this was to reflect the behavior of the stabilization parameter $\beta$ multiplying *C* within the Schur complement approximation. May and Moresi [16] scaled $H=Q$ by the (fixed) viscosity of the fluid; the same scaling is applied in the Cahouet and Chabard preconditioner for generalized Stokes problems [5]. However, none of these investigate the optimal choice of scaling parameter.

Before we continue, let us fix notation. We order the eigenvalues of ${{\mathcal {P}}}_\alpha ^{-1}{{\mathcal {A}}}$ from smallest to largest, so that

1.7 $$\begin{aligned} \lambda _1 \le \dots \le \lambda _{p}< 0 < \lambda _{m+1}\le \dots \le \lambda _{m+n}, \end{aligned}$$

where $p= \text {rank}(S) \le m$ and *S* is in (1.2). Additionally, the notation (*F*, *G*) is used to denote the generalized eigenvalue problem $F{\varvec{v}}= \lambda G{\varvec{v}}$. The $n\times n$ matrix formed by extracting the diagonal of $F\in {\mathbb {R}}^{n\times n}$ will be denoted $\text {diag}(F)$.

## Spectral equivalence bounds

Extensions to existing eigenvalue bounds for the Stokes problem are discussed in this section. We analyse the “ideal” Stokes preconditioner (1.6) first, but we also discuss bounds for efficient “inexact” variants. These results provide informal motivation for modifying the standard saddle-point preconditioner for the Stokes equations. Refined eigenvalue estimates applicable in a Stokes setting are presented in Sect. 3.

### General saddle-point systems

We first wish to fix ideas using a general saddle-point system ${\mathcal {A}}$, preconditioned by ${\mathcal {P}}_{\alpha }$. We characterize the eigenvalues of ${{\mathcal {P}}}_\alpha ^{-1}{{\mathcal {A}}}$ using the following theorem. Although the result is simple, and is similar in flavour to results in many other papers (e.g., [3, 11, 19, 23]), it forms the basis of our analysis and so we provide a proof for completeness. We highlight that this corresponds to an exact application of the (1, 1)-block *A* within the preconditioner.

#### Theorem 2.1

Consider the generalized eigenvalue problem

2.1 $$\begin{aligned} {{\mathcal {A}}}\begin{bmatrix} {{\varvec{x}}}\\ {\varvec{y}}\end{bmatrix} = \lambda {{\mathcal {P}}}_\alpha \begin{bmatrix} {{\varvec{x}}}\\ {\varvec{y}}\end{bmatrix}, \quad {{\mathcal {A}}}= \begin{bmatrix} A&\quad B^T\\ B&\quad -C \end{bmatrix}, \quad {{\mathcal {P}}}_\alpha = \begin{bmatrix} A&\quad 0 \\ 0&\quad \alpha H\end{bmatrix}, \end{aligned}$$

with $A\in {\mathbb {R}}^{n\times n}$ symmetric positive definite, $C\in {\mathbb {R}}^{m\times m}$ symmetric positive semidefinite and $B\in {\mathbb {R}}^{m\times n}$, $m < n$. Assume that $\mathrm{rank}(B) = r \le m$. Then,

I. $\lambda = 1$ with multiplicity $n-r$, with associated eigenvectors $[{{\varvec{x}}}^T,{\varvec{0}}^T]^T$, ${{\varvec{x}}}\in {{\mathrm{null}}}(B)$;
II. $\lambda$ satisfies $-C {\varvec{y}}= \lambda \alpha H{\varvec{y}}$ with ${\varvec{y}}\in {{\mathrm{null}}}(B^T)$, ${\varvec{y}}\ne {\varvec{0}}$, in which case the associated eigenvector of $({{\mathcal {A}}}, {{\mathcal {P}}}_\alpha )$ is $[{\varvec{0}}^T, {\varvec{y}}^T]^T$;
III. or $\lambda = \frac{1}{2}(1-\mu ) \pm \frac{1}{2} \sqrt{(1-\mu )^2 + 4\nu }$, where $\mu = {\varvec{y}}^T C {\varvec{y}}/ {\varvec{y}}^T\alpha H{\varvec{y}}\ge 0$ and $\nu = {\varvec{y}}^T (BA^{-1}B^T+C){\varvec{y}}/{\varvec{y}}^T\alpha H{\varvec{y}}\ge 0$, with ${{\varvec{x}}}\ne {\varvec{0}}$, ${\varvec{y}}\not \in {{\mathrm{null}}}(B^T)$,

where $\lambda \in {\mathbb {R}}$, ${{\varvec{x}}}\in {\mathbb {R}}^n$ and ${\varvec{y}}\in {\mathbb {R}}^m$, with ${{\varvec{x}}}$ and ${\varvec{y}}$ not simultaneously zero vectors. If $C=0$, then Case II occurs if and only if $\lambda = 0$.

#### Proof

Equation (2.1) is equivalent to

2.2 $$\begin{aligned} B^T{\varvec{y}}&= (\lambda -1) A{{\varvec{x}}}, \end{aligned}$$

2.3 $$\begin{aligned} B{{\varvec{x}}}&= (\lambda \alpha H+ C) {\varvec{y}}. \end{aligned}$$

We consider Cases I–III separately.

*Case I * If $\lambda = 1$ then (2.2) implies that $B^T{\varvec{y}}={\varvec{0}}$, so either ${\varvec{y}}={\varvec{0}}$ or ${\varvec{y}}\in {{\mathrm{null}}}(B^T)$, ${\varvec{y}}\ne {\varvec{0}}$. If ${\varvec{y}}= {\varvec{0}}$ then (2.3) implies that $B{{\varvec{x}}}= {\varvec{0}}$, so that ${{\varvec{x}}}\in {{\mathrm{null}}}(B)$. There are $n-r$ linearly independent such vectors. Otherwise, ${\varvec{y}}\in {{\mathrm{null}}}(B^T)$ with ${\varvec{y}}\ne {\varvec{0}}$. However, premultiplying (2.3) by ${\varvec{y}}^T$ then gives that $\alpha {\varvec{y}}^TH{\varvec{y}}= - {\varvec{y}}^TC{\varvec{y}}$. Since $H$ is positive definite, *C* is semidefinite and $\alpha >0$, this cannot hold. Thus, if $\lambda = 1$ then ${\varvec{y}}= {\varvec{0}}$. On the other hand, if ${\varvec{y}}={\varvec{0}}$ we know from (2.2) that $\lambda = 1$, since ${{\varvec{x}}}\ne {\varvec{0}}$ and *A* is positive definite, so $\lambda = 1$ if and only if ${\varvec{y}}= {\varvec{0}}$. Accordingly, 1 is an eigenvalue of $({{\mathcal {A}}},{{\mathcal {P}}}_\alpha )$ with multiplicity $n-r$ and eigenvectors $[{{\varvec{x}}}^T,{\varvec{0}}^T]^T$, ${{\varvec{x}}}\in {{\mathrm{null}}}(B)$.

*Case II * We now assume that ${\varvec{y}}\in {{\mathrm{null}}}(B^T)$, ${\varvec{y}}\ne {\varvec{0}}$. From Case I we know this implies that $\lambda \ne 1$. Then, (2.2) shows that ${{\varvec{x}}}={\varvec{0}}$. From (2.3) it follows that $\lambda$ and ${\varvec{y}}$ satisfy the generalized eigenvalue problem $-C {\varvec{y}}= \lambda \alpha H{\varvec{y}}$. Thus ${\varvec{y}}$ must simultaneously be an eigenvector of $(-C, \alpha H)$ and in the nullspace of $B^T$. At most $m-r$ linearly independent vectors satisfy this requirement. If $C=0$, this case only arises if $\lambda = 0$. On the other hand, if $\lambda =0$ and $C=0$ then (2.2) and (2.3) imply that ${{\varvec{x}}}={\varvec{0}}$ and ${\varvec{y}}\in {{\mathrm{null}}}(B^T)$, so that Case II applies.

*Case III * Otherwise, we know that $\lambda \ne 1$, ${{\varvec{x}}}\ne {\varvec{0}}$, ${\varvec{y}}\not \in {{\mathrm{null}}}(B^T)$. We can rearrange (2.2) for ${{\varvec{x}}}$ and substitute into (2.3) to give

$$\begin{aligned} \frac{1}{\lambda -1} BA^{-1}B^T {\varvec{y}}= (\lambda \alpha H+ C){\varvec{y}}\end{aligned}$$

or $\lambda ^2 - (1-\mu )\lambda - \nu = 0,$ the solution of which is

2.4 $$\begin{aligned} \lambda = \frac{1}{2}(1-\mu ) \pm \frac{1}{2} \sqrt{(1-\mu )^2 + 4\nu } \end{aligned}$$

as required. $$\square $$

We see that it is possible to describe the eigenvalues of ${{\mathcal {P}}}_\alpha ^{-1}{{\mathcal {A}}}$ in terms of *A*, *B*, *C*, *H* and $\alpha$. We also note that when $C=0$ (as arises when solving the Stokes equations using stable finite elements), Case III describes all eigenvalues not equal to 0 or 1.

This is a good place to pause to consider the implications of Theorem 2.1 and the effect of scaling ${{\mathcal {P}}}_\alpha$ on the eigenvalues of the preconditioned matrix for the Stokes equations. Trivially, eigenvalues satisfying Case I are positive (since $\lambda =1$) while any eigenvalues satisfying Case II are non-positive, since *C* is semidefinite and $H$ is positive definite. The remaining eigenvalues of ${{\mathcal {P}}}_\alpha ^{-1}{{\mathcal {A}}}$ may be positive, negative or zero and the inertia of ${{\mathcal {P}}}_\alpha ^{-1}{{\mathcal {A}}}$ must be the same as that of ${{\mathcal {A}}}$. However, because *C* is semidefinite and *A* and $H$ are positive definite, any positive eigenvalue approaches 1 from above as $\alpha$ increases. On the other hand, we see that negative eigenvalues may approach zero from below as $\alpha$ increases, which can have a detrimental affect on the speed of convergence of preconditioned MINRES. For this reason, it is interesting and important to examine in greater detail the effect of $\alpha$ on the eigenvalues of ${{\mathcal {P}}}_\alpha ^{-1}{{\mathcal {A}}}$.

### The Stokes equations

The contributions we provide in this paper rely on the particular numerical properties of the Stokes equations, so we now present a framework for this problem by considering suitable approximations of the (1, 1)-block and associated Schur complement.

We first note that, in practice, an effective preconditioner will not invert the (1, 1)-block exactly as this will be very expensive computationally. However it is reasonable to assume, as in [23], that an approximation ${\widehat{A}}$ may be constructed such that

2.5 $$\begin{aligned} g(h)\le \frac{{\varvec{v}}^{T}A{\varvec{v}}}{{\varvec{v}}^{T}{\widehat{A}}{\varvec{v}}}\le 1,\quad \quad \forall {\varvec{v}}\in {\mathbb {R}}^{n}\backslash \{\varvec{0}\}, \end{aligned}$$

for some function *g* of the mesh parameter *h*. Applying a tailored multigrid method to approximate the action of $A^{-1}$, for example, will achieve this property with *g*(*h*) bounded away from zero independently of *h*. For stable finite element discretizations of the Stokes equation there exists an inf-sup constant $\gamma$, and a constant $\Gamma$ resulting from the boundedness of *B*, such that

2.6 $$\begin{aligned} \gamma ^2\le \frac{{\varvec{p}}^T{}BA^{-1}B^T{\varvec{p}}}{{\varvec{p}}^T{}Q{\varvec{p}}}\le \Gamma ^2,\quad \quad \forall {\varvec{p}}\in {\mathbb {R}}^{m}\backslash \{\varvec{0}\}. \end{aligned}$$

For an unstable discretization only the upper bound holds, and a lower bound is assumed as follows (provided ${\varvec{p}}\notin \text {span}\{\varvec{1}\}$ in the case of enclosed flow):

2.7 $$\begin{aligned} \gamma ^2\le \frac{{\varvec{p}}^T(BA^{-1}B^T+C){\varvec{p}}}{{\varvec{p}}^T{}Q{\varvec{p}}},\quad \frac{{\varvec{p}}^T{}BA^{-1}B^T{\varvec{p}}}{{\varvec{p}}^T{}Q{\varvec{p}}}\le \Gamma ^2,\quad \quad \forall {\varvec{p}}\in {\mathbb {R}}^{m}\backslash \{\varvec{0}\}. \end{aligned}$$

Furthermore we assume that there exist mesh-independent constants $\theta$, $\Theta$ guaranteeing the spectral equivalence of *Q* and the Schur complement approximation *H*, that is:

2.8 $$\begin{aligned} \theta ^2\le \frac{{\varvec{p}}^T{}Q{\varvec{p}}}{{\varvec{p}}^T{}H{\varvec{p}}}\le \Theta ^2,\quad \quad \forall {\varvec{p}}\in {\mathbb {R}}^{m}\backslash \{\varvec{0}\}. \end{aligned}$$

Finally we use the boundedness of *C* to write

2.9 $$\begin{aligned} \frac{{\varvec{p}}^T{}C{\varvec{p}}}{{\varvec{p}}^T{}H{\varvec{p}}}\le \Delta ,\quad \quad \forall {\varvec{p}}\in {\mathbb {R}}^{m}\backslash \{\varvec{0}\}, \end{aligned}$$

for some mesh-independent constant $\Delta$. The properties assumed above all hold for the discretizations and approximations we use in this work. We are now in a position to recall Theorem 2.2 of [23] which in turn provides a bound for the convergence of preconditioned MINRES, see [29, Theorem 4.1].

#### Theorem 2.2

For a stable or stabilized discrete Stokes problem (1.1) on a quasi-uniform sequence of grids, assume that (2.5) holds with $g(h)\rightarrow 0$ as $h\rightarrow 0$, that (2.6) or (2.7) holds, and that (2.8), (2.9) are satisfied. Then the eigenvalues of the preconditioned system $\widehat{{\mathcal {P}}}_{1}^{-1}{\mathcal {A}}$, where

$$\begin{aligned} \widehat{{\mathcal {P}}}_{1}=\begin{bmatrix}{\widehat{A}}&\quad 0 \\ 0&\quad H \end{bmatrix}, \end{aligned}$$

satisfy

$$\begin{aligned} \lambda ({\widehat{{\mathcal {P}}}}_{1}^{-1}{\mathcal {A}})\in {}&\left[ - \Delta /2-\sqrt{\Delta ^2/4+\Gamma ^2\Theta ^2}+{\mathcal {O}}(g(h)),-\gamma \theta \sqrt{g(h)}+{\mathcal {O}}(g(h))\right] \\&\qquad \cup \left[ g(h),1/2+\sqrt{1/4+\Gamma ^2\Theta ^2}\right] . \end{aligned}$$

The asymptotic convergence rate of preconditioned MINRES, denoted by $e_k$, satisfies

2.10 $$\begin{aligned} \lim _{k\rightarrow \infty }~e_k^{1/k}=1-g(h)^{3/4}\sqrt{\frac{4\gamma \theta }{\big ( \Delta +\sqrt{ \Delta ^2+4\Gamma ^2\Theta ^2}\big )\big (1+\sqrt{1+4\Gamma ^2\Theta ^2}\big )}}+{\mathcal {O}}(g(h)^{5/4}). \end{aligned}$$

We refer to [29] for discussion of the asymptotic convergence rate for such problems, and to [26, Chapter 3.2] for a definition and motivation of this quantity. We highlight at this stage that *g*(*h*) is solely determined by how one defines the (1,1) block in the preconditioner, for example using a multigrid method. As methods for approximating such matrices are very well-established, we will therefore regard this as a fixed number. From (2.10) therefore, we observe that the quantity controlling the ‘average’ convergence of the method is

$$\begin{aligned} \mathcal {R}_1:=\frac{4\gamma \theta }{\big (\Delta +\sqrt{\Delta ^2+4\Gamma ^2\Theta ^2}\big )\big (1+\sqrt{1+4\Gamma ^2\Theta ^2}\big )}. \end{aligned}$$

That is, if $\mathcal {R}_1$ is maximized, then the ‘best’ average convergence is achieved. We note that $\Delta =0$ when no stabilization is applied.

#### Remark

In [23, Corollary 2.1], the authors proceed to demonstrate that if ${\widehat{A}}$ is spectrally equivalent to *A* (i.e. with no dependence on *h*), then the convergence rate of the iterative scheme is independent of the mesh. The result (2.10), which assumes some dependence on *h* through the assumption (2.5), does however remain a highly valuable tool to analyse the consequences of parameter changes on the convergence rate, so it is important to state it here.

We should also highlight the fact that the function *g*(*h*) will not depend on *h* in practical applications (since spectrally robust methods such as multigrid can be used to approximate *A*). Therefore the lower bound in assumption (2.5) would ideally be replaced by a mesh-independent constant, $C_{l}$ say. To make this assumption useful in our setting, we may take $g(h)=\min \{C_{l}h^{\delta },C_{l}\}$ for instance, where $\delta >0$ is sufficiently small that $h^{\delta }$ remains roughly 1 for all *h* tested, whereupon (2.5) is satisfied and the dependence on *h* in (2.10) is nullified for all practical computations.

### The effect of scaling

We now consider the result of applying the scaled preconditioner given by

$$\begin{aligned} {\widehat{{\mathcal {P}}}}_{\alpha }=\begin{bmatrix}{\widehat{A}}&\quad 0 \\ 0&\quad \alpha H\end{bmatrix} \end{aligned}$$

on this known theoretical result. Then within our assumptions (2.8), (2.9) for Theorem 2.2, we must replace $\theta ^2$, $\Theta ^2$, $\Delta$ with $\theta ^2/\alpha$, $\Theta ^2/\alpha$, $\Delta /\alpha$, in which case the asymptotic convergence rate becomes

$$\begin{aligned} {\mathcal {R}}_{\alpha }:=\frac{\displaystyle {\frac{4\gamma \theta }{\sqrt{\alpha }}}}{\displaystyle {\Bigg (\frac{\Delta }{\alpha }+\sqrt{\frac{\Delta ^2}{\alpha ^2}+\frac{4\Gamma ^2\Theta ^2}{\alpha }}\Bigg )\Bigg (1+\sqrt{1+\frac{4\Gamma ^2\Theta ^2}{\alpha }}\Bigg )}}. \end{aligned}$$

We now examine the behavior of $\mathcal {R}_{\alpha }$ as $\alpha \uparrow \infty$, starting with the case where a stable discretization is used (i.e. $\Delta =0$). In this case

$$\begin{aligned} {\mathcal {R}}_{\alpha }=\frac{\displaystyle {\frac{4\gamma \theta }{\sqrt{\alpha }}}}{\displaystyle {\frac{2\Gamma \Theta }{\sqrt{\alpha }}\Bigg (1+\sqrt{1+\frac{4\Gamma ^2\Theta ^2}{\alpha }}\Bigg )}}={}&\frac{\displaystyle {\frac{2\gamma \theta }{\Gamma \Theta }}}{\displaystyle {1+\sqrt{1+\frac{4\Gamma ^2\Theta ^2}{\alpha }}}} \end{aligned}$$

so that $\mathcal {R}_{\alpha }\uparrow {}\frac{\gamma \theta }{\Gamma \Theta }$ as $\alpha \uparrow \infty$. In the case where a stabilized mixed method is used (i.e. $\Delta \ne 0$), we have

$$\begin{aligned} \mathcal {R}_{\alpha }={}&\frac{\displaystyle {\frac{4\gamma \theta }{\sqrt{\alpha }}}}{\displaystyle {\Bigg (\frac{\Delta }{\alpha }+\sqrt{\frac{\Delta ^2}{\alpha ^2}+\frac{4\Gamma ^2\Theta ^2}{\alpha }}\Bigg )\Bigg (1+\sqrt{1+\frac{4\Gamma ^2\Theta ^2}{\alpha }}\Bigg )}}\\ ={}&\frac{\displaystyle {4\gamma \theta }}{\displaystyle {\Bigg (\frac{\Delta }{\sqrt{\alpha }}+\sqrt{\frac{\Delta ^2}{\alpha }+4\Gamma ^2\Theta ^2}\Bigg )\Bigg (1+\sqrt{1+\frac{4\Gamma ^2\Theta ^2}{\alpha }}\Bigg )}} \end{aligned}$$

so that $\mathcal {R}_{\alpha } \uparrow {}\frac{\displaystyle {4\gamma \theta }}{\displaystyle {2\Gamma \Theta \cdot 2}}=\frac{\gamma \theta }{\Gamma \Theta }$ as $\alpha \uparrow \infty$.

The above discussion indicates that, for both stable and stabilized discretizations, it may be highly advantageous to increase the scaling parameter $\alpha$ in ${\mathcal {P}}_{\alpha }$. In particular, increasing $\alpha$ nullifies the effect of the parameter $\Delta$ in the expression for the average convergence rate. As $\alpha \uparrow \infty$, the predicted rate tends to $1-g(h)^{3/4}\sqrt{\gamma \theta /\Gamma \Theta }$. Of course this argument is a heuristic, as we do not know from this working how large $\alpha$ must be to result in substantially faster convergence. While scaling a saddle-point preconditioner is a strategy that is commonly adopted by practitioners to accelerate convergence, tuning is invariably done without theoretical justification. We would like to fix this in the Stokes flow context. We provide justification for setting $\alpha$ to a moderately large value in Sects. 3 and 4, and the performance gains that are achievable when choosing a sensible scaling parameter are demonstrated in Sect. 5.

## Refined estimates for the negative eigenvalues of Stokes problems

The bounds in the previous section suggest that large values of $\alpha$ in ${{\mathcal {P}}}_\alpha$ will reduce the condition number of ${{\mathcal {P}}}_\alpha ^{-1}{{\mathcal {A}}}$ and hence improve the convergence rate of preconditioned MINRES applied to Stokes problems. Fast convergence of Krylov subspace methods for symmetric indefinite problems is often attributed to nicely distributed eigenvalues, with clustered eigenvalues often sought.^4^ Recalling the remarks after Theorem 2.1, we find that positive eigenvalues of ${{\mathcal {P}}}_\alpha ^{-1}{{\mathcal {A}}}$ cluster near one as $\alpha$ increases. Negative eigenvalues also cluster as $\alpha$ increases, but move towards the origin, which can delay the convergence of Krylov subspace methods.

Accordingly, it is instructive to more precisely characterize $\lambda _{p}$, the negative eigenvalue of ${{\mathcal {P}}}_\alpha ^{-1}{{\mathcal {A}}}$ nearest the origin, for Stokes problems discretized by different finite elements. In particular, we examine stable $Q_2 \textendash Q_1$ elements, and the two stabilized elements available in the Incompressible Flow & Iterative Solver Software (IFISS) [9, 10, 22] software. These are $Q_1 \textendash Q_1$ elements with the stabilization approach of Dohrmann and Bochev [7] (see also [8, Sect. 3.3.2]) and $Q_1 \textendash P_0$ elements stabilized as in [8, Section 3.3.2]. Note that for these $Q_1 \textendash P_0$ elements the pressure mass matrix is diagonal, so that $Q = \text {diag}(Q)$. We assume in this section that the (1, 1) block of ${{\mathcal {P}}}_\alpha$ is *A* and the (2, 2) block is either the pressure mass matrix or its diagonal.

To motivate our analysis, we compute the extreme negative and positive eigenvalues $\lambda _1$, $\lambda _{p}$, $\lambda _{m+1}$ and $\lambda _{m+n}$ of ${{\mathcal {P}}}_\alpha ^{-1}{{\mathcal {A}}}$ as $\alpha$ varies, for a cavity problem discretized by $Q_1 \textendash Q_1$, $Q_1 \textendash P_0$ and $Q_2 \textendash Q_1$ elements. This is a widely considered problem in Stokes flow, which we define on $\Omega =[-1,1]^{2}$, with $\vec {f}=\vec {0}$ and boundary conditions given by

$$\begin{aligned} \ v_{x}=1-x^{4},~v_{y}=0,\quad&\text {on }[-1,1]\times \{1\}, \\ \ v_{x}=v_{y}=0,\quad&\text {on }\partial \Omega \backslash \big ([-1,1]\times \{1\}\big ), \end{aligned}$$

where $\vec {v}=[v_{x},~v_{y}]^{T}$. Since the flow is enclosed, the preconditioned system is singular with a single zero eigenvalue that is associated with a zero velocity and a constant pressure vector.

Tables 1 and 2 are consistent with the asymptotic results for large $\alpha$ in Sect. 2. We also note that $\lambda _{p}$ approaches the origin algebraically as $\alpha$ is increased. Other interesting trends also emerge. One intriguing feature of $Q_1 \textendash Q_1$ elements is that, when *H* in ${{\mathcal {P}}}_\alpha$ is the diagonal of the pressure mass matrix, the eigenvalue $\lambda _{p}$ seems to be $-0.25/\alpha$. On the other hand, when *H* is the full pressure mass matrix and $\alpha$ is large the eigenvalue $\lambda _{p}$ is almost (although not exactly) the same for all three element types.

Our next task is to develop good bounds for $\lambda _p$ and explain some of the phenomena we observe, so that we might choose a value of $\alpha$ that results in fast convergence of Krylov methods applied to Stokes problems. To do this we examine both Case II and Case III eigenvalues from Theorem 2.1.

**Table 1:** Computed extreme eigenvalues of ${{\mathcal {P}}}_\alpha ^{-1}{{\mathcal {A}}}$ for the cavity problem, a mesh parameter of $2^{-5}$ and $H = \text {diag}(Q)$, the diagonal of the pressure mass matrix

$\alpha$	$Q_1 \textendash Q_1$			$Q_2 \textendash Q_1$		
	$\lambda _{1}$	$\lambda _{p}$	$\lambda _{m+n}$	$\lambda _{1}$	$\lambda _{p}$	$\lambda _{m+n}$
1	$-1.1\times 10^{0}$	$-2.5\times 10^{-1}$	2.1	$-1.1\times 10^{0}$	$-1.1\times 10^{-1}$	2.1
2	$-6.7\times 10^{-1}$	$-1.2\times 10^{-1}$	1.7	$-6.5\times 10^{-1}$	$-6.0\times 10^{-2}$	1.7
3	$-5.0\times 10^{-1}$	$-8.3\times 10^{-2}$	1.5	$-4.9\times 10^{-1}$	$-4.1\times 10^{-2}$	1.5
4	$-4.0\times 10^{-1}$	$-6.3\times 10^{-2}$	1.4	$-3.9\times 10^{-1}$	$-3.1\times 10^{-2}$	1.4
5	$-3.3\times 10^{-1}$	$-5.0\times 10^{-2}$	1.3	$-3.3\times 10^{-1}$	$-2.5\times 10^{-2}$	1.3
6	$-2.9\times 10^{-1}$	$-4.2\times 10^{-2}$	1.3	$-2.8\times 10^{-1}$	$-2.1\times 10^{-2}$	1.3
7	$-2.5\times 10^{-1}$	$-3.6\times 10^{-2}$	1.3	$-2.5\times 10^{-1}$	$-1.8\times 10^{-2}$	1.2
8	$-2.3\times 10^{-1}$	$-3.1\times 10^{-2}$	1.2	$-2.2\times 10^{-1}$	$-1.6\times 10^{-2}$	1.2
9	$-2.1\times 10^{-1}$	$-2.8\times 10^{-2}$	1.2	$-2.0\times 10^{-1}$	$-1.4\times 10^{-2}$	1.2
10	$-1.9\times 10^{-1}$	$-2.5\times 10^{-2}$	1.2	$-1.8\times 10^{-1}$	$-1.3\times 10^{-2}$	1.2
20	$-1.0\times 10^{-1}$	$-1.2\times 10^{-2}$	1.1	$-9.9\times 10^{-2}$	$-6.3\times 10^{-3}$	1.1
40	$-5.3\times 10^{-2}$	$-6.2\times 10^{-3}$	1.1	$-5.1\times 10^{-2}$	$-3.2\times 10^{-3}$	1.1
60	$-3.6\times 10^{-2}$	$-4.2\times 10^{-3}$	1.0	$-3.5\times 10^{-2}$	$-2.1\times 10^{-3}$	1.0
80	$-2.7\times 10^{-2}$	$-3.1\times 10^{-3}$	1.0	$-2.6\times 10^{-2}$	$-1.6\times 10^{-3}$	1.0
100	$-2.2\times 10^{-2}$	$-2.5\times 10^{-3}$	1.0	$-2.1\times 10^{-2}$	$-1.3\times 10^{-3}$	1.0

[In each case, $\lambda _{m+1}=1$ to at least 3 significant figures.]

**Table 2:** Computed extreme eigenvalues of ${{\mathcal {P}}}_\alpha ^{-1}{{\mathcal {A}}}$ for the cavity problem, a mesh parameter of $2^{-5}$ and $H = Q$, the pressure mass matrix

$\alpha$	$Q_1 \textendash Q_1$			$Q_1 \textendash P_0$			$Q_2 \textendash Q_1$		
	$\lambda _{1}$	$\lambda _{p}$	$\lambda _{m+n}$	$\lambda _{1}$	$\lambda _{p}$	$\lambda _{m+n}$	$\lambda _{1}$	$\lambda _{p}$	$\lambda _{m+n}$
1	$-1.1\times 10^{0}$	$-1.9\times 10^{-1}$	1.6	$-1.3\times 10^{0}$	$-2.0\times 10^{-1}$	1.6	$-6.2\times 10^{-1}$	$-1.8\times 10^{-1}$	1.6
2	$-5.5\times 10^{-1}$	$-1.0\times 10^{-1}$	1.4	$-7.2\times 10^{-1}$	$-1.1\times 10^{-1}$	1.4	$-3.7\times 10^{-1}$	$-9.5\times 10^{-2}$	1.4
3	$-3.8\times 10^{-1}$	$-7.1\times 10^{-2}$	1.3	$-5.0\times 10^{-1}$	$-7.3\times 10^{-2}$	1.3	$-2.6\times 10^{-1}$	$-6.5\times 10^{-2}$	1.3
4	$-2.9\times 10^{-1}$	$-5.4\times 10^{-2}$	1.2	$-3.9\times 10^{-1}$	$-5.5\times 10^{-2}$	1.2	$-2.1\times 10^{-1}$	$-4.9\times 10^{-2}$	1.2
5	$-2.3\times 10^{-1}$	$-4.4\times 10^{-2}$	1.2	$-3.1\times 10^{-1}$	$-4.5\times 10^{-2}$	1.2	$-1.7\times 10^{-1}$	$-4.0\times 10^{-2}$	1.2
6	$-2.0\times 10^{-1}$	$-3.7\times 10^{-2}$	1.1	$-2.7\times 10^{-1}$	$-3.8\times 10^{-2}$	1.1	$-1.5\times 10^{-1}$	$-3.3\times 10^{-2}$	1.1
7	$-1.7\times 10^{-1}$	$-3.2\times 10^{-2}$	1.1	$-2.3\times 10^{-1}$	$-3.2\times 10^{-2}$	1.1	$-1.3\times 10^{-1}$	$-2.9\times 10^{-2}$	1.1
8	$-1.5\times 10^{-1}$	$-2.8\times 10^{-2}$	1.1	$-2.0\times 10^{-1}$	$-2.8\times 10^{-2}$	1.1	$-1.1\times 10^{-1}$	$-2.5\times 10^{-2}$	1.1
9	$-1.3\times 10^{-1}$	$-2.5\times 10^{-2}$	1.1	$-1.8\times 10^{-1}$	$-2.5\times 10^{-2}$	1.1	$-1.0\times 10^{-1}$	$-2.3\times 10^{-2}$	1.1
10	$-1.2\times 10^{-1}$	$-2.2\times 10^{-2}$	1.1	$-1.6\times 10^{-1}$	$-2.3\times 10^{-2}$	1.1	$-9.2\times 10^{-2}$	$-2.0\times 10^{-2}$	1.1
20	$-6.1\times 10^{-2}$	$-1.1\times 10^{-2}$	1.0	$-8.5\times 10^{-2}$	$-1.2\times 10^{-2}$	1.0	$-4.8\times 10^{-2}$	$-1.0\times 10^{-2}$	1.0
40	$-3.1\times 10^{-2}$	$-5.7\times 10^{-3}$	1.0	$-4.3\times 10^{-2}$	$-5.8\times 10^{-3}$	1.0	$-2.4\times 10^{-2}$	$-5.2\times 10^{-3}$	1.0
60	$-2.1\times 10^{-2}$	$-3.8\times 10^{-3}$	1.0	$-2.9\times 10^{-2}$	$-3.9\times 10^{-3}$	1.0	$-1.6\times 10^{-2}$	$-3.4\times 10^{-3}$	1.0
80	$-1.5\times 10^{-2}$	$-2.8\times 10^{-3}$	1.0	$-2.2\times 10^{-2}$	$-2.9\times 10^{-3}$	1.0	$-1.2\times 10^{-2}$	$-2.6\times 10^{-3}$	1.0
100	$-1.2\times 10^{-2}$	$-2.3\times 10^{-3}$	1.0	$-1.7\times 10^{-2}$	$-2.3\times 10^{-3}$	1.0	$-9.9\times 10^{-3}$	$-2.1\times 10^{-3}$	1.0

[In each case, $\lambda _{m+1}=1$ to at least 3 significant figures.]

### Case III eigenvalues

We start by studying Lemma 2.3 in [23] (adapted to include $\alpha$), which can also be obtained by bounding the Case III eigenvalues in Theorem 2.1. Importantly, when $H=Q$, the pressure mass matrix, the bound is remarkably tight. Although the same bound can be applied when $H=\text {diag}(Q)$, we will see that the results are less informative since $\theta ^2\gamma ^2$ is far from $\nu _{\min }$, the smallest value of $\nu$ in Theorem 2.1.

#### Lemma 3.1

[23, Lemma 2.3], [8, Theorem 4.7] For the discrete stable or stabilized Stokes problem (1.1) on a quasi-uniform sequence of grids, assume that (2.6) or (2.7) hold and that (2.8) and (2.9) are satisfied. Then

3.1 $$\begin{aligned} \lambda _{p}\le \frac{1}{2}\left( 1 - \sqrt{1+4\theta ^2\gamma ^2/\alpha }\right) . \end{aligned}$$

Since $Q_1 \textendash Q_1$ and $Q_1 \textendash P_0$ elements satisfy the ideal stabilization property (see [8, Sect. 3.3.2]), the largest eigenvalue of $Q^{-1}C$ is less than or equal to 1 for these elements. Additionally, for stable $Q_2 \textendash Q_1$ elements $C=0$ so that (2.9) is trivially satisfied. Thus, for all three elements we can apply Lemma 3.1. Moreover, $\gamma$ is bounded away from zero by a constant that depends on the element type but not on the mesh parameter *h* [8, Sect. 3.5]. The smallest value, $\nu _{\min }$, of $\nu$ in Theorem 2.1 approximates $\gamma ^2$ and is given in Table 3 for the cavity problem; for the obstacle problem this is introduced in Sect. 5.

**Table 3:** Values of $\nu _{\min }$, the smallest value of $\nu$ in Theorem 2.1, for different problems and element types when the mesh parameter is $2^{-5}$ and $H=Q$

	$Q_1 \textendash Q_1$	$Q_1 \textendash P_0$	$Q_2 \textendash Q_1$
Regularized cavity	$2.386\times 10^{-1}$	$2.339\times 10^{-1}$	$2.074\times 10^{-1}$
Obstacle	$8.776\times 10^{-3}$	$8.692\times 10^{-3}$	$8.771\times 10^{-3}$

Tables 4 and 5 show the bound (3.1) and corresponding value of $\lambda _{p}$ for the cavity problem. We see that the bound is pessimistic when $H = \text {diag}(Q)$ (with the exception of $Q_1 \textendash P_0$ elements for which $\text {diag}(Q)=Q$). However, the bound is very accurate for all three elements when the full pressure mass matrix is used in ${{\mathcal {P}}}_\alpha$. Additionally, when $H=Q$, it appears that the eigenvalue $\lambda _{p}$ is determined mainly by $\nu _{\min }$, which varies only mildly between the different element types, and which is bounded away from zero independently of *h*. Qualitatively similar results are observed for the obstacle problem described in Sect. 5. Importantly, it seems that when $H$ is the pressure mass matrix we can accurately bound $\lambda _{p}$ as $\alpha$ increases, which allows us to control the magnitude of this eigenvalue.

**Table 4:** Largest negative eigenvalue ($\lambda _{p}$) of ${{\mathcal {P}}}_\alpha ^{-1}{{\mathcal {A}}}$, and bound (3.1) for the cavity problem, a mesh parameter of $2^{-5}$ and $H = \text {diag}(Q)$, the diagonal of the pressure mass matrix

$\alpha$	$Q_1 \textendash Q_1$		$Q_2 \textendash Q_1$	
	$\lambda _{p}$	Bound	$\lambda _{p}$	Bound
1	$-2.5\times 10^{-1}$	$-5.6\times 10^{-2}$	$-1.1\times 10^{-1}$	$-4.9\times 10^{-2}$
2	$-1.2\times 10^{-1}$	$-2.9\times 10^{-2}$	$-6.0\times 10^{-2}$	$-2.5\times 10^{-2}$
3	$-8.3\times 10^{-2}$	$-2.0\times 10^{-2}$	$-4.1\times 10^{-2}$	$-1.7\times 10^{-2}$
4	$-6.3\times 10^{-2}$	$-1.5\times 10^{-2}$	$-3.1\times 10^{-2}$	$-1.3\times 10^{-2}$
5	$-5.0\times 10^{-2}$	$-1.2\times 10^{-2}$	$-2.5\times 10^{-2}$	$-1.0\times 10^{-2}$
6	$-4.2\times 10^{-2}$	$-9.8\times 10^{-3}$	$-2.1\times 10^{-2}$	$-8.6\times 10^{-3}$
7	$-3.6\times 10^{-2}$	$-8.4\times 10^{-3}$	$-1.8\times 10^{-2}$	$-7.4\times 10^{-3}$
8	$-3.1\times 10^{-2}$	$-7.4\times 10^{-3}$	$-1.6\times 10^{-2}$	$-6.4\times 10^{-3}$
9	$-2.8\times 10^{-2}$	$-6.6\times 10^{-3}$	$-1.4\times 10^{-2}$	$-5.7\times 10^{-3}$
10	$-2.5\times 10^{-2}$	$-5.9\times 10^{-3}$	$-1.3\times 10^{-2}$	$-5.2\times 10^{-3}$
20	$-1.2\times 10^{-2}$	$-3.0\times 10^{-3}$	$-6.3\times 10^{-3}$	$-2.6\times 10^{-3}$
40	$-6.2\times 10^{-3}$	$-1.5\times 10^{-3}$	$-3.2\times 10^{-3}$	$-1.3\times 10^{-3}$
60	$-4.2\times 10^{-3}$	$-9.9\times 10^{-4}$	$-2.1\times 10^{-3}$	$-8.6\times 10^{-4}$
80	$-3.1\times 10^{-3}$	$-7.5\times 10^{-4}$	$-1.6\times 10^{-3}$	$-6.5\times 10^{-4}$
100	$-2.5\times 10^{-3}$	$-6.0\times 10^{-4}$	$-1.3\times 10^{-3}$	$-5.2\times 10^{-4}$

**Table 5:** Largest negative eigenvalue ($\lambda _{p}$) of ${{\mathcal {P}}}_\alpha ^{-1}{{\mathcal {A}}}$, and bound (3.1) for the cavity problem, a mesh parameter of $2^{-5}$ and $H = Q$, the pressure mass matrix

$\alpha$	$Q_1 \textendash Q_1$		$Q_1 \textendash P_0$		$Q_2 \textendash Q_1$	
	$\lambda _{p}$	Bound	$\lambda _{p}$	Bound	$\lambda _{p}$	Bound
1	$-1.9\times 10^{-1}$	$-2.0\times 10^{-1}$	$-2.0\times 10^{-1}$	$-2.0\times 10^{-1}$	$-1.8\times 10^{-1}$	$-1.8\times 10^{-1}$
2	$-1.0\times 10^{-1}$	$-1.1\times 10^{-1}$	$-1.1\times 10^{-1}$	$-1.1\times 10^{-1}$	$-9.5\times 10^{-2}$	$-9.5\times 10^{-2}$
3	$-7.1\times 10^{-2}$	$-7.4\times 10^{-2}$	$-7.3\times 10^{-2}$	$-7.3\times 10^{-2}$	$-6.5\times 10^{-2}$	$-6.5\times 10^{-2}$
4	$-5.4\times 10^{-2}$	$-5.6\times 10^{-2}$	$-5.5\times 10^{-2}$	$-5.5\times 10^{-2}$	$-4.9\times 10^{-2}$	$-4.9\times 10^{-2}$
5	$-4.4\times 10^{-2}$	$-4.6\times 10^{-2}$	$-4.5\times 10^{-2}$	$-4.5\times 10^{-2}$	$-4.0\times 10^{-2}$	$-4.0\times 10^{-2}$
6	$-3.7\times 10^{-2}$	$-3.8\times 10^{-2}$	$-3.8\times 10^{-2}$	$-3.8\times 10^{-2}$	$-3.3\times 10^{-2}$	$-3.3\times 10^{-2}$
7	$-3.2\times 10^{-2}$	$-3.3\times 10^{-2}$	$-3.2\times 10^{-2}$	$-3.2\times 10^{-2}$	$-2.9\times 10^{-2}$	$-2.9\times 10^{-2}$
8	$-2.8\times 10^{-2}$	$-2.9\times 10^{-2}$	$-2.8\times 10^{-2}$	$-2.8\times 10^{-2}$	$-2.5\times 10^{-2}$	$-2.5\times 10^{-2}$
9	$-2.5\times 10^{-2}$	$-2.6\times 10^{-2}$	$-2.5\times 10^{-2}$	$-2.5\times 10^{-2}$	$-2.3\times 10^{-2}$	$-2.3\times 10^{-2}$
10	$-2.2\times 10^{-2}$	$-2.3\times 10^{-2}$	$-2.3\times 10^{-2}$	$-2.3\times 10^{-2}$	$-2.0\times 10^{-2}$	$-2.0\times 10^{-2}$
20	$-1.1\times 10^{-2}$	$-1.2\times 10^{-2}$	$-1.2\times 10^{-2}$	$-1.2\times 10^{-2}$	$-1.0\times 10^{-2}$	$-1.0\times 10^{-2}$
40	$-5.7\times 10^{-3}$	$-5.9\times 10^{-3}$	$-5.8\times 10^{-3}$	$-5.8\times 10^{-3}$	$-5.2\times 10^{-3}$	$-5.2\times 10^{-3}$
60	$-3.8\times 10^{-3}$	$-4.0\times 10^{-3}$	$-3.9\times 10^{-3}$	$-3.9\times 10^{-3}$	$-3.4\times 10^{-3}$	$-3.4\times 10^{-3}$
80	$-2.8\times 10^{-3}$	$-3.0\times 10^{-3}$	$-2.9\times 10^{-3}$	$-2.9\times 10^{-3}$	$-2.6\times 10^{-3}$	$-2.6\times 10^{-3}$
100	$-2.3\times 10^{-3}$	$-2.4\times 10^{-3}$	$-2.3\times 10^{-3}$	$-2.3\times 10^{-3}$	$-2.1\times 10^{-3}$	$-2.1\times 10^{-3}$

### Case II eigenvalues

Although the eigenvalue bound (3.1) is descriptive when we use the full pressure mass matrix in ${{\mathcal {P}}}_\alpha$, it is rather pessimistic when we use its diagonal instead (except for $Q_1 \textendash P_0$ elements). It would be useful to have an alternative means of quantifying $\lambda _{p}$, when $H=\text {diag}(Q)$, for $Q_1 \textendash Q_1$ and $Q_2 \textendash Q_1$ elements. The latter case appears to be difficult, since there are no Case II eigenvalues in Theorem 2.1. However, we see from Table 1 that for $Q_1 \textendash Q_1$ elements $\lambda _{p}$ behaves like $-0.25/\alpha$. We show in the rest of this section that this is indeed the case, and that this eigenvalue is associated with Case II in Theorem 2.1. Since it is possible to characterize the Case II eigenvalues for the full pressure mass matrix, and for $Q_1 \textendash P_0$ elements, we extend our analysis to these cases for completeness.

**Fig. 1:**
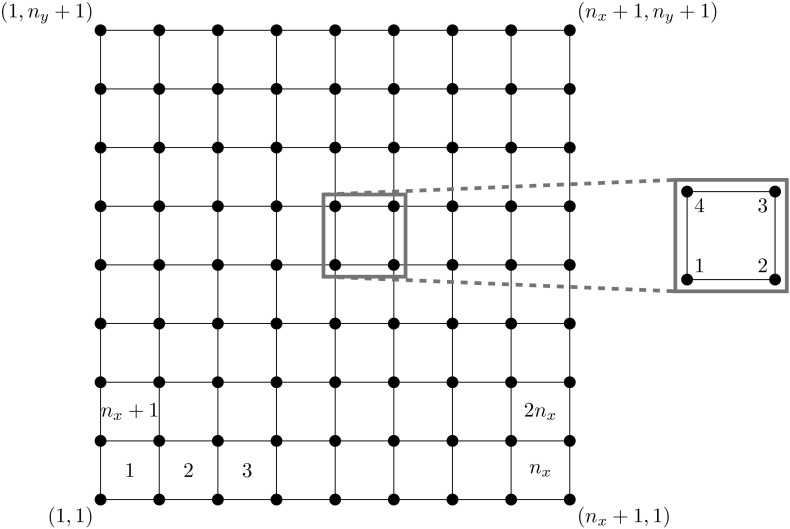
Diagram of mesh and nodes (*left*), and node numbering within each element (*right*)

Case II eigenvalues satisfy $-C {\varvec{y}}= \lambda \alpha H{\varvec{y}}$, ${\varvec{y}}\in {{\mathrm{null}}}(B^T)$. Our approach for this analysis is to propose a basis for ${{\mathrm{null}}}(B^T)$, and then determine whether these basis vectors are eigenvectors of the generalized problem $(-C, \alpha H)$. To do so we require certain notation, and details of the finite element assembly process, that we describe here. We assume that there are $n_x$ elements in the *x* direction and $n_y$ elements in the *y* direction, so that the total number of elements is $n_{el} = n_xn_y$ (see Fig. 1). Although we restrict our attention to rectangular domains for simplicity, the same methodology can be used to analyse more complicated domains, as we discuss at the end of this section.

Let us first briefly introduce some notation to describe the assembly process. Let $C_k\in {\mathbb {R}}^{\ell \times \ell }$, $Q_k\in {\mathbb {R}}^{\ell \times \ell }$ and $\text {diag}(Q_k)\in {\mathbb {R}}^{\ell \times \ell }$, $k = 1,\cdots , n_{el}$, be the element matrices that are assembled to form *C*, *Q* and $\text {diag}(Q)$. Additionally, let $L\in {\mathbb {R}}^{N\times m}$ be the connectivity matrix that maps local pressure degrees of freedom on element *k* to the global pressure degrees of freedom $1,\cdots ,m$, where $N= \ell n_{el}$. Then

3.2 $$\begin{aligned} C = L^T\text {diag}(C_k)L,~~Q = L^T \text {diag}(Q_k) L,~~\text {diag}(Q)= L^T \text {diag}(\text {diag}(Q_k)) L.\qquad \end{aligned}$$

#### $Q_1 \textendash Q_1$ elements

We now examine Case II eigenvalues for $Q_1 \textendash Q_1$ elements. Let us begin by specifying the $Q_1 \textendash Q_1$ connectivity matrix.

##### Lemma 3.2

Let the Stokes equations be discretized by $Q_1 \textendash Q_1$ elements on a rectangular domain of square elements, with $n_x$ elements in one direction and $n_y$ elements in the other. Let $L\in {\mathbb {R}}^{N\times m}$ be the $Q_1 \textendash Q_1$ connectivity matrix that maps the $N = 4n_xn_y$ local pressure degrees of freedom to the *m* global pressure degrees of freedom. Consider the (*i*, *j*)th node in the finite element mesh, where the node number is as in Fig. 1. Then the corresponding column of *L* is given by

3.3 $$\begin{aligned} \ell _k = {\left\{ \begin{array}{ll} {\varvec{e}}_1\otimes {\varvec{e}}_1\otimes {\varvec{e}}_1 &{} i = 1, j = 1,\\ {\varvec{e}}_1\otimes \big [ {\varvec{e}}_{i-1}\otimes {\varvec{e}}_2 + {\varvec{e}}_i\otimes {\varvec{e}}_1\big ] &{} i = 2,\cdots ,n_x, j = 1,\\ {\varvec{e}}_1\otimes {\varvec{e}}_{n_x}\otimes {\varvec{e}}_2 &{} i = n_x+1, j = 1,\\ {\varvec{e}}_{j-1}\otimes {\varvec{e}}_1\otimes {\varvec{e}}_4 + {\varvec{e}}_{j}\otimes {\varvec{e}}_1\otimes {\varvec{e}}_1 &{} i = 1, j = 2,\cdots ,n_y,\\ {\varvec{e}}_{j-1}\otimes \big [{\varvec{e}}_{i-1}\otimes {\varvec{e}}_3 + {\varvec{e}}_{i}\otimes {\varvec{e}}_4 \big ] &{} \\ \quad \quad + {\varvec{e}}_{j}\otimes \big [{\varvec{e}}_{i-1}\otimes {\varvec{e}}_2 + {\varvec{e}}_i\otimes {\varvec{e}}_1\big ] &{} i = 2,\cdots ,n_x, j = 2,\cdots ,n_y,\\ {\varvec{e}}_{j-1}\otimes {\varvec{e}}_{n_x}\otimes {\varvec{e}}_3 + {\varvec{e}}_{j}\otimes {\varvec{e}}_{n_x}\otimes {\varvec{e}}_2 &{} i = n_x+1, j = 2,\cdots ,n_y,\\ {\varvec{e}}_{n_y}\otimes {\varvec{e}}_1\otimes {\varvec{e}}_4 &{} i = 1, j = n_y+1,\\ {\varvec{e}}_{n_y}\otimes \big [{\varvec{e}}_{i-1}\otimes {\varvec{e}}_3 + {\varvec{e}}_i\otimes {\varvec{e}}_4\big ] &{} i = 2,\cdots ,n_k, j = n_y+1,\\ {\varvec{e}}_{n_y}\otimes {\varvec{e}}_{n_x}\otimes {\varvec{e}}_3 &{} i = n_x+1, j = n_y+1, \end{array}\right. }\quad \end{aligned}$$

with $k = (j-1)(n_x+1) + i$, $i = 1, \cdots , n_x+1$ and $j = 1,\cdots , n_y+1$. In each Kronecker product ${\varvec{e}}_j \otimes {\varvec{e}}_i \otimes {\varvec{e}}_s$, the vectors ${\varvec{e}}_j\in {\mathbb {R}}^{n_y}$, ${\varvec{e}}_i\in {\mathbb {R}}^{n_x}$ and ${\varvec{e}}_s\in {\mathbb {R}}^4$ are the *j*th, *i*th and $s$th unit vectors of the appropriate dimension.

Since *L* has one element per row,

3.4 $$\begin{aligned} L{\varvec{1}}_m = {\varvec{1}}_{N}, \end{aligned}$$

that is, the connectivity matrix maps the constant vector to one of larger dimension (cf. Lemma 3.4 below).

As stated at the start of this section, we employ the stabilization approach of Dohrmann and Bochev, who define the stabilization matrix on the *k*th element to be

3.5 $$\begin{aligned} C_k = Q_k - {\varvec{q}}{\varvec{q}}^T |\square _k|, \end{aligned}$$

where ${\varvec{q}}= [\frac{1}{4},\frac{1}{4},\frac{1}{4},\frac{1}{4}]^T$ and $|\square _k|$ is the element area. With this choice it follows from (3.2) that the stabilization matrix *C* satisfies $C = Q - |\square _k|{\varvec{w}}{\varvec{w}}^T,$ where ${\varvec{w}}= \frac{1}{4}L^T {\varvec{1}}_{N}$. It is straightforward to compute that ${{\mathrm{null}}}(C_k)= {{\mathrm{span}}}\{{\varvec{1}}_4\}$. Since the connectivity matrix maps the constant vector to one of larger dimension (see (3.4)), ${{\mathrm{null}}}(C) = {{\mathrm{span}}}\{{\varvec{1}}_m\}$.

Recall that Case II eigenvalues satisfy $- C {\varvec{y}}= \lambda \alpha H{\varvec{y}}$, ${\varvec{y}}\in {{\mathrm{null}}}(B^T)$. Without any modifications to *B* to incorporate essential boundary conditions, ${{\mathrm{null}}}(B^T) = \mathrm{span}\{\varvec{\pm 1}_m \}$, where $\varvec{\pm 1}$ is the vector of alternating signs, i.e. $(\varvec{\pm 1})_k = (-1)^{k+1}$ [20, 21]. Imposing essential boundary conditions may increase the dimension of ${{\mathrm{null}}}(B^T)$.

To show that we have a Case II eigenvalue, we must be able to show that $\varvec{\pm 1}$ is an eigenvector of $- C {\varvec{y}}= \lambda \alpha \,\text {diag}(Q){\varvec{y}}$ or, equivalently, of

$$\begin{aligned} {\varvec{0}}= L^T (C_k + \lambda \alpha \,\text {diag}(Q_k))L {\varvec{y}}. \end{aligned}$$

Since the eigenvalues of $(-C,\alpha \,\text {diag}(Q))$ are closely related to those of $(-C_k,\alpha \,\text {diag}(Q_k))$, we first determine the eigenvalues and eigenvectors of this small problem.

##### Lemma 3.3

Let the Stokes equations be discretized by $Q_1 \textendash Q_1$ elements on a rectangular domain of square elements. The eigenpairs of $(-C_k,\alpha \,\mathrm{diag}(Q_k))$ are $(\theta _s,{\widetilde{{\varvec{v}}}}_s)$, $s=1,\cdots ,4$, where

$$\begin{aligned} \Theta =\begin{bmatrix}\theta _1&&\\&\theta _2&\\&\theta _3&\\&&\theta _4\end{bmatrix} = \begin{bmatrix} 0&&\\&-\frac{0.25}{\alpha }&\\&-\frac{0.75}{\alpha }&\\&&-\frac{0.75}{\alpha }\end{bmatrix} \end{aligned}$$

and

$$\begin{aligned} V = \begin{bmatrix}{\widetilde{{\varvec{v}}}}_1&{\widetilde{{\varvec{v}}}}_2&{\widetilde{{\varvec{v}}}}_3&{\widetilde{{\varvec{v}}}}_4\end{bmatrix} = \left[ \begin{array}{r r r r} 1 &{}\quad -1 &{}\quad 1 &{}\quad 1\\ 1 &{}\quad 1 &{}\quad 1 &{}\quad -1\\ 1 &{}\quad -1 &{}\quad -1 &{}\quad -1\\ 1 &{}\quad 1 &{}\quad -1 &{}\quad 1\end{array}\right] . \end{aligned}$$

##### Proof

The result is obtained by straightforward computation. $\square$

The eigenpair $(\theta _2,{\widetilde{{\varvec{v}}}}_2)$ seems promising since $\theta _2 = -0.25/\alpha$ matches the observed value of $\lambda _{p}$ in Table 1, while ${\widetilde{{\varvec{v}}}}_2 = \varvec{\pm 1}_4$. To find the corresponding eigenpairs of $(-C, \alpha \,\text {diag}(Q))$ we now extend ${\widetilde{{\varvec{v}}}}_s$, $s= 1,\cdots ,4$, to vectors of length *m* via

3.6 $$\begin{aligned} {\varvec{v}}_s= (L^TL)^{-1}L^T {\widehat{{\varvec{v}}}}_s, \end{aligned}$$

where

3.7 $$\begin{aligned} {\widehat{{\varvec{v}}}}_s= {\left\{ \begin{array}{ll} {\varvec{1}}_{n_y}\otimes {\varvec{1}}_{n_x}\otimes {\widetilde{{\varvec{v}}}}_1 = {\varvec{1}}&{} s= 1,\\ \varvec{\pm 1}_{n_y}\otimes \varvec{\pm 1}_{n_x}\otimes {\widetilde{{\varvec{v}}}}_2 = \varvec{\pm 1}&{} s= 2,\\ \varvec{\pm 1}_{n_y}\otimes {\varvec{1}}_{n_x}\otimes {\widetilde{{\varvec{v}}}}_3 &{} s= 3,\\ {\varvec{1}}_{n_y}\otimes \varvec{\pm 1}_{n_x}\otimes {\widetilde{{\varvec{v}}}}_4 &{} s= 4. \end{array}\right. } \end{aligned}$$

Note that

3.8 $$\begin{aligned} {\widehat{{\varvec{v}}}}_s= [\epsilon _1 {\widetilde{{\varvec{v}}}}_s^T, \epsilon _2 {\widetilde{{\varvec{v}}}}_s^T, \cdots , \epsilon _{n_{el}}{\widetilde{{\varvec{v}}}}_s^T ]^T, \end{aligned}$$

with $$\epsilon _k \in \{-1,1 \}$$, $$k = 1,\cdots ,n_{el}$$.

To proceed we require a technical result that shows that the ${\widehat{{\varvec{v}}}}_s$ lie in ${{\mathrm{range}}}(L)$.

##### Lemma 3.4

The vectors ${\widehat{{\varvec{v}}}}_s$, $s= 1,\cdots ,4$ in (3.7) satisfy ${\widehat{{\varvec{v}}}}_s\in {{\mathrm{range}}}(L)$, where *L* is the $Q_1 \textendash Q_1$ connectivity matrix in (3.3).

##### Proof

We must be able to combine columns $\ell _p$ of *L* in (3.3) to get ${\widehat{{\varvec{v}}}}_s$, $s= 1,\cdots ,4$. It is straightforward, although rather cumbersome, to show that

$$\begin{aligned} {\widehat{{\varvec{v}}}}_1&= \sum _{j = 1}^{n_y+1}\sum _{i = 1}^{n_x+1} \ell _{(j-1)(n_x+1)+i},&{\widehat{{\varvec{v}}}}_2&= \sum _{j = 1}^{n_y+1}\sum _{i = 1}^{n_x+1} (-1)^{i+j}\ell _{(j-1)(n_x+1)+i},\\ {\widehat{{\varvec{v}}}}_3&= \sum _{j = 1}^{n_y+1}\sum _{i = 1}^{n_x+1} (-1)^{i+1}\ell _{(j-1)(n_x+1)+i},&{\widehat{{\varvec{v}}}}_4&= \sum _{j = 1}^{n_y+1}\sum _{i = 1}^{n_x+1} (-1)^{j+1}\ell _{(j-1)(n_x+1)+i}, \end{aligned}$$

which proves the result. $\square$

Since $L(L^TL)^{-1}L^T$ is an orthogonal projector onto ${{\mathrm{range}}}(L)$, a consequence of Lemma 3.4 is that

3.9 $$\begin{aligned} L{\varvec{v}}_s= L(L^TL)^{-1}L^T{\widehat{{\varvec{v}}}}_s= {\widehat{{\varvec{v}}}}_s,\quad s= 1,\cdots ,4 . \end{aligned}$$

Importantly, this means that $L{\varvec{v}}_2 = {\widehat{{\varvec{v}}}}_2 = \varvec{\pm 1}\in {{\mathrm{null}}}(B^T)$.

The final step is to combine (3.9) with Lemma 3.3 to show that ${\widehat{{\varvec{v}}}}_2$ is indeed an eigenvector of $(-C,\alpha \,\text {diag}(Q))$, with corresponding eigenvalue $-0.25/\alpha$.

##### Lemma 3.5

Let the Stokes equations be discretized by $Q_1 \textendash Q_1$ elements on a rectangular domain of square elements. The pairs $(\lambda _s, {\varvec{v}}_s)$, $s= 1,\cdots ,4$ satisfy $-C{\varvec{v}}_s= \lambda _s\alpha \,\mathrm{diag}(Q) {\varvec{v}}_s$, where $\lambda _s$ are as in Lemma 3.3 and ${\varvec{v}}_s$ are defined by (3.6).

##### Proof

From (3.2) we have that $C{\varvec{v}}_s+ \lambda _s\alpha \,\text {diag}(Q){\varvec{v}}_s= L^T(C_k + \lambda _s\alpha \,\text {diag}(Q_k))L{\varvec{v}}_s$. Thus, using (3.8), (3.9) and Lemma 3.3, we find that

$$\begin{aligned}&C{\varvec{v}}_s+ \lambda _s\alpha \,\text {diag}(Q_k){\varvec{v}}_s\\&\quad =L^T\begin{bmatrix}C_k + \lambda _s\alpha \,\text {diag}(Q_k)&&\\&C_k + \lambda _s\alpha \,\text {diag}(Q_k)&\\&\ddots&\\&&C_k + \lambda _s\alpha \,\text {diag}(Q_k)\end{bmatrix} \begin{bmatrix} \epsilon _1 {\widetilde{{\varvec{v}}}}_s\\ \epsilon _2 {\widetilde{{\varvec{v}}}}_s\\ \vdots \\ \epsilon _{n_{el}}{\widetilde{{\varvec{v}}}}_s\end{bmatrix}\\&\quad ={\varvec{0}}, \end{aligned}$$

which shows that $(\lambda _s, {\varvec{v}}_s)$ are eigenpairs of $(-C,\alpha \,\text {diag}(Q))$. $\square$

Now we are in a position to determine Case II eigenvalues.

##### Theorem 3.6

Let the Stokes equations in two dimensions be discretized on a rectangular domain with square elements by $Q_1 \textendash Q_1$ elements, and let $H = \mathrm{diag}(Q)$. Then $-0.25/\alpha$ is the largest negative Case II eigenvalue of ${{\mathcal {P}}}_\alpha ^{-1}{{\mathcal {A}}}$.

##### Proof

The vectors ${\varvec{v}}_s$, $s= 1,\cdots ,4$ are candidates for ${\varvec{y}}$ in Case II eigenvalues in Theorem 2.1. As discussed above ${\varvec{v}}_2$ lies in ${{\mathrm{null}}}(B^T)$ before *B* is modified to accommodate any essential boundary conditions [20, 21]. Thus $-0.25/\alpha$ is an eigenvalue of ${{\mathcal {P}}}_\alpha ^{-1}{{\mathcal {A}}}$.

Our final task is to show that $-0.25/\alpha$ is the largest negative Case II eigenvalue. To do so we employ the approach of Wathen [28]. We let $\widetilde{Q}_k$ represent the diagonal matrix whose diagonal entries are those of $Q_k$ to simplify notation. Since $\varvec{1}_m = L \varvec{1}_{N}$, $N = 4n_{el}$, any nonzero Case II eigenvalue $\lambda$ must satisfy

$$\begin{aligned} \lambda&\le -\frac{1}{\alpha } \min _{\begin{array}{c} {{\varvec{x}}}\ne 0\\ {{\varvec{x}}}\perp \varvec{1}_m \end{array}} \frac{{{\varvec{x}}}^T C {{\varvec{x}}}}{{{\varvec{x}}}^T\,\text {diag}(Q){{\varvec{x}}}}\\&= -\frac{1}{\alpha } \min _{\begin{array}{c} {{\varvec{x}}}\ne 0\\ {{\varvec{x}}}\perp \varvec{1}_m \end{array}} \frac{{{\varvec{x}}}^T L^T \text {diag}(C_k) L {{\varvec{x}}}}{{{\varvec{x}}}^TL^T \text {diag}(\widetilde{Q}_k) L{{\varvec{x}}}} \le -\frac{1}{\alpha } \min _{\begin{array}{c} {\varvec{y}}\ne 0\\ {\varvec{y}}\perp \varvec{1}_N \end{array}} \frac{{\varvec{y}}^T \text {diag}(C_k){\varvec{y}}}{{\varvec{y}}^T \text {diag}(\widetilde{Q}_k) {\varvec{y}}} = -\frac{0.25}{\alpha }. \end{aligned}$$

$$\square $$

The eigenvalue $\lambda = -0.25/\alpha$ is thus precisely $\lambda _{p}$ that we observe in Table 1. Of course, certain boundary conditions may increase the dimension of ${{\mathrm{null}}}(B^T)$, in which case ${\varvec{v}}_1$, ${\varvec{v}}_3$ and/or ${\varvec{v}}_4$ may lie in the nullspace of this modified matrix. For example, for the channel problem all four vectors lie in the nullspace.

For completeness we now consider the case where $H= Q$, the pressure mass matrix, which can be analysed in a very similar manner to $H= \text {diag}(Q)$ above.

##### Theorem 3.7

Let the Stokes equations in two dimensions be discretized on a rectangular domain with square elements by $Q_1 \textendash Q_1$ elements, and let $H = Q$. Then $\lambda = -1/\alpha$ is the largest Case II eigenvalue of ${{\mathcal {P}}}_\alpha ^{-1}{{\mathcal {A}}}$.

##### Proof

If $- C {\varvec{v}}= \lambda \alpha Q {\varvec{v}}$ then ${\varvec{0}}= L^T (C_k + \lambda \alpha Q_k)L {\varvec{v}}.$ The four eigenpairs of $(-C_k,\alpha Q_k)$ are $(0,{\widetilde{{\varvec{v}}}}_1)$ and $(-1/\alpha ,{\widetilde{{\varvec{v}}}}_s)$, $s = 2,3,4$. A result similar to Lemma 3.5 then shows that $(0,{\varvec{v}}_1)$, $(-1/\alpha , {\varvec{v}}_s)$, $s= 2,3,4$, are eigenpairs of $(-C,\alpha Q)$.

As in the proof of Theorem 3.6, before *B* is modified to accommodate boundary conditions, ${{\mathrm{null}}}(B^T) = \mathrm{span}\{{\varvec{v}}_2 \}$ and the eigenvalue $-1/\alpha$ is guaranteed to be a Case II eigenvalue of ${{\mathcal {P}}}_\alpha ^{-1}{{\mathcal {A}}}$.

A similar generalized Rayleigh-quotient analysis to that in the proof of Lemma 3.6 shows that this is the most negative Case II eigenvalue. $\square$

Again, ${\varvec{v}}_1$, ${\varvec{v}}_3$ and/or ${\varvec{v}}_4$ may lie in the nullspace of *B* after essential boundary conditions are imposed. We stress that the difference between this and the diagonal pressure mass matrix approximation is that $\lambda _{p}\ne -1/\alpha$. Instead $\lambda _{p}$ is as in Table 2, and is bounded by (3.1).

More generally, we can bound the largest Case II eigenvalues for any preconditioner that is spectrally equivalent to *Q*, i.e., any preconditioner for which (2.8) holds.

##### Corollary 3.8

Let the Stokes equations in two dimensions be discretized on a rectangular domain with square elements by $Q_1 \textendash Q_1$ elements, and let *H* satisfy (2.8). Then the largest nonzero Case II eigenvalue of ${{\mathcal {P}}}_\alpha ^{-1}{{\mathcal {A}}}$ is bounded above by $-\theta ^2/\alpha$.

##### Proof

Any nonzero Case II eigenvalue satisfies

$$\begin{aligned} \lambda \le -\frac{1}{\alpha } \min _{\begin{array}{c} {{\varvec{x}}}\ne 0\\ {{\varvec{x}}}\perp \varvec{1}_m \end{array}} \frac{{{\varvec{x}}}^T C {{\varvec{x}}}}{{{\varvec{x}}}^T H {{\varvec{x}}}} \le -\frac{1}{\alpha } \min _{\begin{array}{c} {{\varvec{x}}}\ne 0\\ {{\varvec{x}}}\perp \varvec{1}_m \end{array}} \frac{{{\varvec{x}}}^T C {{\varvec{x}}}}{{{\varvec{x}}}^T Q {{\varvec{x}}}} \min _{{{\varvec{x}}}\ne 0} \frac{{{\varvec{x}}}^T Q {{\varvec{x}}}}{{{\varvec{x}}}^T H {{\varvec{x}}}} = -\frac{\theta ^2}{\alpha }, \end{aligned}$$

where we have used Theorem 3.7. $\square$

Corollary 3.8 allows us to bound Case II eigenvalues for general preconditioners. Moreover, it gives some insight into whether it is likely that $\lambda _p$ is a Case II eigenvalue or a Case III eigenvalue.

#### $Q_1 \textendash P_0$ elements

We now turn our attention to $Q_1 \textendash P_0$ elements, which have one pressure degree of freedom per element, located at the element centre. A consequence is that the pressure mass matrix is diagonal, so that $Q = \text {diag}(Q)= |\square _k|I$, where $|\square _k|$ is the area of a single element. The stabilization matrix we choose is that in [8, Sect. 3.3.2], which we briefly describe here. Consider a macroelement comprising a $2\times 2$ patch of elements. Then the *k*th macroelement stabilization matrix is

$$\begin{aligned} C_k = |\square _k|\left[ \begin{array}{r r r r} 2 &{}\quad -1 &{}\quad 0 &{}\quad -1\\ -1 &{}\quad 2 &{}\quad -1 &{}\quad 0\\ 0 &{}\quad -1 &{}\quad 2 &{}\quad -1\\ -1 &{}\quad 0 &{}\quad -1 &{}\quad 2\end{array}\right] . \end{aligned}$$

Additionally, $Q_k = |\square _k|I$, and the connectivity matrix that maps pressure degrees of freedom on a macroelement to global degrees of freedom is the identity, i.e. $L=I$.

For these elements $B^T$ has full rank except in the case of Dirichlet boundary conditions, in which case ${{\mathrm{null}}}(B^T) = \mathrm{span}\{{\varvec{v}}_1, {\varvec{v}}_2\}$ where ${\varvec{v}}_1$ and ${\varvec{v}}_2$ are as in (3.7).

##### Theorem 3.9

Let the Stokes equations in two dimensions be discretized on a rectangular domain with square elements by $Q_1 \textendash P_0$ elements, and let $H = Q$. If Dirichlet conditions are imposed on the whole boundary then 0 and $-1/\alpha$ are both Case II eigenvalues of ${{\mathcal {P}}}_\alpha ^{-1}{{\mathcal {A}}}$. Otherwise, there are no Case II eigenvalues.

##### Proof

It is straightforward to compute that $(C_k,Q_k)$ has eigenpairs $(0,{\widetilde{{\varvec{v}}}}_1)$, $(4,{\widetilde{{\varvec{v}}}}_2)$, $(2,{\widetilde{{\varvec{v}}}}_3)$ and $(2,{\widetilde{{\varvec{v}}}}_4)$, where ${\widetilde{{\varvec{v}}}}_s$, $s= 1,\cdots ,4$, are as in Lemma 3.3. Since

$$\begin{aligned} C {\varvec{v}}- \lambda Q{\varvec{v}}= \text {diag}(C_k - \lambda Q_k){\varvec{v}}, \end{aligned}$$

the results of Sect. 3.2.1 can be applied to show that $(0,{\varvec{v}}_1)$, $(4,{\varvec{v}}_2)$, $(2,{\varvec{v}}_3)$ and $(2,{\varvec{v}}_4)$ are all eigenpairs of (*C*, *Q*). In fact, because *C* is block diagonal and *Q* is diagonal, it is possible to take $\varvec{e}_j \otimes {\varvec{v}}_s$, $j = 1,\cdots ,n_{el}$, as eigenvectors, where $\varvec{e}_j\in {\mathbb {R}}^{n_{el}}$ is the *j*th unit vector. Thus, for problems with purely Dirichlet boundary conditions, ${\varvec{v}}_1$ and ${\varvec{v}}_4$ lie in ${{\mathrm{null}}}(B^T$), and both $(0,{\varvec{v}}_4)$ and $(1,{\varvec{v}}_1)$ are Case II eigenpairs. Otherwise, there are no Case II eigenvalues. $\square$

Similarly to $Q_1 \textendash Q_1$ elements, we can bound Case II eigenvalues for any preconditioner that satisfies (2.8).

##### Corollary 3.10

Let the Stokes equations in two dimensions be discretized on a rectangular domain with square elements by $Q_1 \textendash P_0$ elements, and let *H* satisfy (2.8). If Dirichlet conditions are imposed on the whole boundary then nonzero Case II eigenvalues are bounded above by $-\theta ^2/\alpha$. Otherwise, there are no Case II eigenvalues.

##### Proof

The proof is analogous to that of Corollary 3.8. $\square$

### Possible extensions

It is clear that the methodology outlined above could be applied to non-square domains to ascertain the presence of Case II eigenvalues, although it may be more difficult to determine the appropriate nullspace vectors [20], and the connectivity matrix may be more complicated to describe.

As an example of what we might expect for more general domains, we performed numerical experiments on the L-shaped domain for the backward-facing step problem in IFISS (see Sect. 3.1 of [8] for a full problem description). We numerically verified that when $Q_1 \textendash Q_1$ elements are used, $(-C,\alpha \,\text {diag}(Q))$ has eigenpairs $(0,{\varvec{v}}_1)$, $(-0.25/\alpha , {\varvec{v}}_2)$, $(-0.75/\alpha , {\varvec{v}}_3)$ and $(-0.75/\alpha , {\varvec{v}}_4)$, while $(-C,\alpha Q)$ has eigenpairs $(0,{\varvec{v}}_1)$ and $(-1/\alpha , {\varvec{v}}_s)$, $s= 2,3,4$, i.e. the same eigenpairs as for the square domain. Since ${\varvec{v}}_2\in {{\mathrm{null}}}(B^T)$, $-0.25/\alpha$ is a Case II eigenvalue. Moreover, after boundary conditions are applied we find that $(-0.75/\alpha , {\varvec{v}}_4)$ is an additional Case II eigenvalue. For $Q_1 \textendash P_0$ elements we find that $(0,{\varvec{v}}_1)$, $(-4/\alpha ,{\varvec{v}}_2)$, $(-2/\alpha ,{\varvec{v}}_3)$ and $(-2/\alpha ,{\varvec{v}}_4)$ are eigenpairs of $(-C,\alpha Q)$. However, because this problem has a natural outflow condition there are no Case II eigenpairs. We note that exactly the same results hold for the obstacle problem described in the next section.

Although in this section we used the ideal preconditioner ${{\mathcal {P}}}_\alpha$, additional numerical experiments (described in Sect. 5), that are conducted with *A* replaced by a single V-cycle of algebraic multigrid (AMG), show that only $\lambda _{m+1}$, which takes values between 0.84 and 0.94, changes significantly when this approximation is made. Additionally, we note that we could replace $\text {diag}(Q)$, the diagonal of the mass matrix, by ${{\mathrm{lump}}}(Q)$, the lumped mass matrix whose entries are the row sums of *Q*, or by a fixed number of iterations of Chebyshev semi-iteration. Both approaches cause $\lambda _1$, $\lambda _{p}$, $\lambda _{m+1}$ and $\lambda _{m+n}$ to better approximate the values obtained when $H = Q$. In particular, the analysis in this section could be straightforwardly adapted for lumped mass matrices; this is particularly easy for the $Q_1$ pressure mass matrices considered here for which ${{\mathrm{lump}}}(Q)= 2.25\,\text {diag}(Q)$.

### Summary and interpretation

Theorem 2.1 and the subsequent analysis tells us that increasing the parameter $\alpha$ in ${\mathcal {P}}_{\alpha }$ leads to more clustered eigenvalues of ${\mathcal {P}}_{\alpha }^{-1}{\mathcal {A}}$ for a range of Stokes problems, and should result in more rapid convergence of the MINRES algorithm. The likely drawback of this was that the negative eigenvalues of the preconditioned system could approach zero at a rapid rate as $\alpha$ is increased—in this section we have shown that this does not occur.

A key question is therefore how the value of $\alpha$ should be selected for practical computations. Although the theory suggests there is no “optimal” choice, we believe that a reasonable selection, and one that fits with the desire for the eigenvalues of ${\mathcal {P}}_{\alpha }^{-1}{\mathcal {A}}$ to be bounded away from zero, is one that ensures that ${\mathcal {P}}_{\alpha }$ is as well conditioned as possible. It is well known [8, Chapter 1] that the eigenvalues of the pressure mass matrix are contained within $[c_{m}h^{\bar{d}},C_{m}h^{\bar{d}}]$, and those of the stiffness matrix within $[c_{a}h^{\bar{d}},C_{a}h^{\bar{d}-2}]$, for positive *h*-independent constants $c_{m}$, $C_{m}$, $c_{a}$, $C_{a}$, and with $\bar{d}$ the dimension of the problem. Therefore, when seeking the best possible conditioning of ${\mathcal {P}}_{\alpha }$, by “balancing” the blocks ${\widehat{A}}$ and $\alpha {}H$, it important to ensure that the parameter $\alpha$ does not exceed an ${\mathcal {O}}(h^{-2})$ value. In practice, we find that a much more moderate scaling, such as ${\mathcal {O}}(10)$, is sufficient to ensure more rapid convergence of the MINRES algorithm for a range of Stokes problems.

## MINRES convergence bounds for Stokes problems

In the previous two sections we characterized the effects of $\alpha$ on the eigenvalues of ${{\mathcal {P}}}_\alpha ^{-1}{{\mathcal {A}}}$. It is now of interest to ascertain the effect of varying $\alpha$ on the number of iterations required for preconditioned MINRES to converge to a fixed tolerance.

The following MINRES convergence bounds are well known (see, e.g., [8, Sect. 4.2.4]):

4.1 $$\begin{aligned} \frac{\Vert {\varvec{r}}_{k}\Vert _{{{\mathcal {P}}}_\alpha ^{-1}}}{\Vert {\varvec{r}}_{0}\Vert _{{{\mathcal {P}}}_\alpha ^{-1}}} \le \min _{\begin{array}{c} p\in \Pi _k\\ p(0)=1 \end{array}} \max _{\lambda \in \sigma ({{\mathcal {P}}}_\alpha ^{-1}{{\mathcal {A}}})} |p(\lambda )| \le \min _{\begin{array}{c} p\in \Pi _k\\ p(0)=1 \end{array}} \max _{\lambda \in [-a,-b]\cup [c,d]} |p(\lambda )|, \end{aligned}$$

where $\Pi _k$ is the set of polynomials of at most degree *k* and $\sigma ({{\mathcal {P}}}_\alpha ^{-1}{{\mathcal {A}}}) \subset [-a,-b]\cup [c,d]$ is the set of nonzero eigenvalues of ${{\mathcal {P}}}_\alpha ^{-1}{{\mathcal {A}}}$. Note that for enclosed flow problems, which give singular but consistent systems, convergence is affected only by nonzero eigenvalues of ${{\mathcal {P}}}_\alpha ^{-1}{{\mathcal {A}}}$ [8, Sect. 2.3], [25, Chapter 10]. This polynomial approximation problem is difficult to solve, in general. The exception is if $a-b = d-c$, i.e. the two intervals are of equal length, in which case [8, Sect. 4.2.4]:

4.2 $$\begin{aligned} \frac{\Vert {\varvec{r}}_{2k}\Vert _{{{\mathcal {P}}}_\alpha ^{-1}}}{\Vert {\varvec{r}}_{0}\Vert _{{{\mathcal {P}}}_\alpha ^{-1}}} \le 2 \eta ^k, \quad \eta = \frac{\sqrt{ad} - \sqrt{bc}}{\sqrt{ad} + \sqrt{bc}} . \end{aligned}$$

Although this bound can be pessimistic, it will still provide some insight into the effect of $\alpha$ on preconditioned MINRES convergence.

### Lemma 4.1

Let the Stokes equations be discretized by $Q_2 \textendash Q_1$ elements in ${\mathbb {R}}^2$, and assume that (2.6) holds. Let ${{\mathcal {P}}}_\alpha$ be as in (1.6). Then, the eigenvalues of ${{\mathcal {P}}}_\alpha ^{-1}{{\mathcal {A}}}$ are contained in $[-a,-b] \cup \{0\} \cup [c,d]$ where, if $H = Q$,

$$\begin{aligned} 2a = \sqrt{1 + \frac{4\Phi }{\alpha }}-1, \quad 2b = \sqrt{1+\frac{4\gamma ^2}{\alpha }}-1, \quad c = 1, \quad 2d = 1+ \sqrt{1 + \frac{4\Phi }{\alpha }}. \end{aligned}$$

Alternatively, if $H = \mathrm{diag}(Q)$ then

$$\begin{aligned} 2a = \sqrt{1 + \frac{9\Phi }{\alpha }}-1, \quad 2b = \sqrt{1+\frac{\gamma ^2}{\alpha }}-1, \quad c = 1, \quad 2d = 1 + \sqrt{1 + \frac{9\Phi }{\alpha }}. \end{aligned}$$

Here, $\gamma$ is the inf-sup constant in (2.6), while $\Phi = 1$ if Dirichlet conditions are imposed on the whole boundary and $\Phi = 2$ otherwise.

### Proof

The bounds for $H=Q$ can be obtained from Theorem 2.1 and Lemma 3.1, noting that for stable elements $C = 0$, $\mu = 0$, and there are no Case II eigenvalues in Theorem 2.1. We note that the parameter $\nu$ defined in Theorem 2.1 satisfies $\nu \le \Phi /\alpha$ [8, Eqs. (3.164) and (3.169)].

The bounds for $H = \text {diag}(Q)$ are obtained similarly. The only additional step is in bounding $\nu$. Since, for all ${\varvec{y}}\in {\mathbb {R}}^m$, ${\varvec{y}}\ne \varvec{0}$, it holds that [28]

4.3 $$\begin{aligned} \frac{1}{4}\le \frac{{\varvec{y}}^T Q {\varvec{y}}}{{\varvec{y}}^T\,\text {diag}(Q){\varvec{y}}} \le \frac{9}{4}, \end{aligned}$$

it follows that

$$\begin{aligned} \nu \le \max _{\begin{array}{c} {\varvec{y}}\in {\mathbb {R}}^m\\ {\varvec{y}}\not \in {{\mathrm{null}}}(B^T) \end{array}} \frac{{\varvec{y}}^T (BA^{-1}B^T + C){\varvec{y}}}{{\varvec{y}}^T \alpha Q {\varvec{y}}} \frac{{\varvec{y}}^T Q {\varvec{y}}}{{\varvec{y}}^T\,\text {diag}(Q){\varvec{y}}} \le \frac{\Phi }{\alpha } \frac{9}{4}. \end{aligned}$$

Using this inequality gives the required bounds. $\square$

### Lemma 4.2

Let the Stokes equations be discretized by $Q_1 \textendash Q_1$ or $Q_1 \textendash P_0$ elements in ${\mathbb {R}}^2$, and assume that (2.7) holds. Let ${{\mathcal {P}}}_\alpha$ be as in (1.6). Then the eigenvalues of ${{\mathcal {P}}}_\alpha ^{-1}{{\mathcal {A}}}$ are contained in $[-a,-b] \cup \{0\} \cup [c,d]$ where, if $H = Q$,

$$\begin{aligned} 2a= & {} \sqrt{\left( 1-\frac{1}{\alpha }\right) ^2 + \frac{4\Phi }{\alpha }}\, - \left( 1-\frac{1}{\alpha }\right) , \quad 2b = \sqrt{1+\frac{4\gamma ^2}{\alpha }}-1, \quad c = 1,\\ 2d= & {} 1 +\sqrt{1 + \frac{4\Phi }{\alpha }}. \end{aligned}$$

Alternatively, for $Q_1 \textendash Q_1$ elements if $H = \mathrm{diag}(Q)$ then, assuming that $\lambda _{p}= -0.25/\alpha$,

$$\begin{aligned} 2a = \sqrt{\left( 1-\frac{9}{4\alpha }\right) ^2 + \frac{9\Phi }{\alpha }}- \left( 1-\frac{9}{4\alpha }\right) , \quad b = \frac{0.25}{\alpha }, \quad c = 1, \quad 2d = 1 + \sqrt{1 + \frac{9\Phi }{\alpha }}. \end{aligned}$$

Here, $\gamma$ is as in (2.7) while $\Phi = 2$ if Dirichlet conditions are imposed on the whole boundary and $\Phi = 3$ otherwise.

### Proof

Let us start with $H=Q$. Both *c* and *d* follow from Theorem 2.1 (Cases I and III), noting that $\nu \le \Phi /\alpha$ [8, Eqs. (3.164) and (3.169)]. Additionally, Lemma 3.1 shows that all negative eigenvalues in Theorem 2.1 are bounded above by $-b$.

To show that all eigenvalues are bounded below by $-a$, first note that since $Q_1 \textendash P_0$ and $Q_1 \textendash Q_1$ elements satisfy the ideal stabilization property, the largest eigenvalue of $Q^{-1}C$ is less than or equal to 1. Thus, no Case II eigenvalue is smaller than $-1/\alpha$. Since

$$\begin{aligned} -a \le \frac{1}{2}\left( 1-\frac{1}{\alpha }\right) - \frac{1}{2}\sqrt{\left( 1-\frac{1}{\alpha }\right) ^2 + \frac{4}{\alpha }} = -\frac{1}{\alpha }, \end{aligned}$$

Case II eigenvalues are no smaller than $-a$. It is straightforward to show that Case III eigenvalues are also bounded below by $-a$.

If $H = \text {diag}(Q)$ the proof is similar to the above if we again use (4.3) to replace *Q* by $\text {diag}(Q)$ in $\nu$ and $\mu$. $\square$

To assess the effect of $\alpha$ on (4.2), and hence on the convergence rate of preconditioned MINRES, we compute *a*, *b*, *c*, *d* using Lemma 4.1 or 4.2 (see Tables 6 and 7). Comparison with Tables 1 and 2 shows that the eigenvalue bounds for $Q_2 \textendash Q_1$ elements are very tight. For the stabilized elements *a* and *d* overestimate the magnitude of the extreme eigenvalues, but in almost all cases only by a small amount. The exception is *a* for $Q_1 \textendash Q_1$ elements, which is close to twice $|\lambda _1|$.

**Fig. 2:**
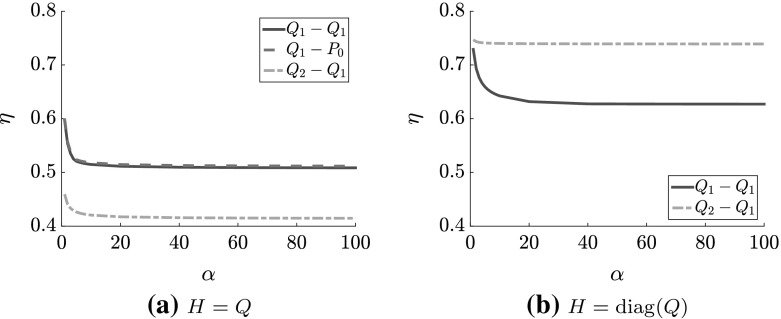
$\eta$ in (4.2) for the cavity problem, with a mesh parameter of $2^{-5}$

We then increase *a* or *d* so that both intervals $[-a,-b]$ and [*c*, *d*] are of equal length, and apply (4.2). The results, in Fig. 2, clearly show that increasing $\alpha$ reduces $\eta$, but that as we increase $\alpha$ beyond about 10, $\eta$ decreases much more slowly. In other words, as $\alpha$ is increased beyond this point, we would not anticipate a further significant reduction in iteration numbers for our preconditioned solver. This therefore motivates a value of $\alpha$ equal to roughly 10, as this choice essentially achieves the optimal predicted convergence rate, while at the same time ensuring that the negative eigenvalues of ${\mathcal {P}}_{\alpha }^{-1}{\mathcal {A}}$ are far from zero. This pattern of behavior will be realized in numerical experiments discussed in the next section.

**Table 6:** Eigenvalue bounds from Lemmas 4.1 and 4.2 for the cavity problem with a mesh parameter of $2^{-5}$ and $H = Q$, the pressure mass matrix

$\alpha$	$Q_1 \textendash Q_1$			$Q_1 \textendash P_0$			$Q_2 \textendash Q_1$		
	*a*	*b*	*d*	*a*	*b*	*d*	*a*	*b*	*d*
1	$1.4\times 10^{0}$	$2.0\times 10^{-1}$	2.0	$1.4\times 10^{0}$	$2.0\times 10^{-1}$	2.0	$6.2\times 10^{-1}$	$1.8\times 10^{-1}$	1.6
2	$7.8\times 10^{-1}$	$1.1\times 10^{-1}$	1.6	$7.8\times 10^{-1}$	$1.1\times 10^{-1}$	1.6	$3.7\times 10^{-1}$	$9.5\times 10^{-2}$	1.4
3	$5.5\times 10^{-1}$	$7.4\times 10^{-2}$	1.5	$5.5\times 10^{-1}$	$7.3\times 10^{-2}$	1.5	$2.6\times 10^{-1}$	$6.5\times 10^{-2}$	1.3
4	$4.3\times 10^{-1}$	$5.6\times 10^{-2}$	1.4	$4.3\times 10^{-1}$	$5.5\times 10^{-2}$	1.4	$2.1\times 10^{-1}$	$4.9\times 10^{-2}$	1.2
5	$3.5\times 10^{-1}$	$4.6\times 10^{-2}$	1.3	$3.5\times 10^{-1}$	$4.5\times 10^{-2}$	1.3	$1.7\times 10^{-1}$	$4.0\times 10^{-2}$	1.2
6	$3.0\times 10^{-1}$	$3.8\times 10^{-2}$	1.3	$3.0\times 10^{-1}$	$3.8\times 10^{-2}$	1.3	$1.5\times 10^{-1}$	$3.3\times 10^{-2}$	1.1
7	$2.6\times 10^{-1}$	$3.3\times 10^{-2}$	1.2	$2.6\times 10^{-1}$	$3.2\times 10^{-2}$	1.2	$1.3\times 10^{-1}$	$2.9\times 10^{-2}$	1.1
8	$2.3\times 10^{-1}$	$2.9\times 10^{-2}$	1.2	$2.3\times 10^{-1}$	$2.8\times 10^{-2}$	1.2	$1.1\times 10^{-1}$	$2.5\times 10^{-2}$	1.1
9	$2.0\times 10^{-1}$	$2.6\times 10^{-2}$	1.2	$2.0\times 10^{-1}$	$2.5\times 10^{-2}$	1.2	$1.0\times 10^{-1}$	$2.3\times 10^{-2}$	1.1
10	$1.8\times 10^{-1}$	$2.3\times 10^{-2}$	1.2	$1.8\times 10^{-1}$	$2.3\times 10^{-2}$	1.2	$9.2\times 10^{-2}$	$2.0\times 10^{-2}$	1.1
20	$9.6\times 10^{-2}$	$1.2\times 10^{-2}$	1.1	$9.6\times 10^{-2}$	$1.2\times 10^{-2}$	1.1	$4.8\times 10^{-2}$	$1.0\times 10^{-2}$	1.0
40	$4.9\times 10^{-2}$	$5.9\times 10^{-3}$	1.0	$4.9\times 10^{-2}$	$5.8\times 10^{-3}$	1.0	$2.4\times 10^{-2}$	$5.2\times 10^{-3}$	1.0
60	$3.3\times 10^{-2}$	$4.0\times 10^{-3}$	1.0	$3.3\times 10^{-2}$	$3.9\times 10^{-3}$	1.0	$1.6\times 10^{-2}$	$3.4\times 10^{-3}$	1.0
80	$2.5\times 10^{-2}$	$3.0\times 10^{-3}$	1.0	$2.5\times 10^{-2}$	$2.9\times 10^{-3}$	1.0	$1.2\times 10^{-2}$	$2.6\times 10^{-3}$	1.0
100	$2.0\times 10^{-2}$	$2.4\times 10^{-3}$	1.0	$2.0\times 10^{-2}$	$2.3\times 10^{-3}$	1.0	$9.9\times 10^{-3}$	$2.1\times 10^{-3}$	1.0

We end this section by discussing the effect of $\alpha$ on the norm used to measure convergence of preconditioned MINRES. A common stopping criterion is a specified reduction in the preconditioned residual norm, i.e. given a symmetric positive definite preconditioner ${\mathcal {P}}$ we terminate when $\Vert {\varvec{r}}_k\Vert _{{\mathcal {P}}^{-1}}/\Vert {\varvec{r}}_0\Vert _{{\mathcal {P}}^{-1}}<\tau$, where

$$\begin{aligned} {\varvec{r}}_k = \begin{bmatrix}{\varvec{r}}_k^{(1)}\\{\varvec{r}}_k^{(2)}\end{bmatrix} = \begin{bmatrix}\varvec{f}\\{\varvec{g}}\end{bmatrix} -\begin{bmatrix}A&\quad B^T\\B&\quad -C\end{bmatrix} \begin{bmatrix}{\varvec{x}}_k^{(1)}\\{\varvec{x}}_k^{(2)}\end{bmatrix} \end{aligned}$$

is the *k*th residual and $[ ({\varvec{x}}_k^{(1)})^T ~ ({\varvec{x}}_k^{(2)})^T]^T$ is the *k*th preconditioned MINRES iterate. It is straightforward to see that

$$\begin{aligned} \Vert {\varvec{r}}_k^{ }\Vert _{{\mathcal {P}}_\alpha ^{-1}}^2 = \Vert {\varvec{r}}_k^{(1)}\Vert _{A^{-1}_{ }}^2+ \alpha ^{-1}\Vert {\varvec{r}}_k^{(2)}\Vert _{H^{-1}_{ }}^2. \end{aligned}$$

In this sense increasing $\alpha$ relaxes the stopping criterion for the constraint equation $B{\varvec{x}} = {\varvec{g}}$. In our experience this is not a problem, as we show in the next section.

**Table 7:** Eigenvalue bounds from Lemmas 4.1 and 4.2 for the cavity problem with a mesh parameter of $2^{-5}$ and $H = \text {diag}(Q)$, the diagonal of the pressure mass matrix

$\alpha$	$Q_1 \textendash Q_1$			$Q_2 \textendash Q_1$		
	*a*	*b*	*d*	*a*	*b*	*d*
1	$2.8\times 10^{0}$	$2.5\times 10^{-1}$	2.7	$1.1\times 10^{0}$	$4.9\times 10^{-2}$	2.1
2	$1.6\times 10^{0}$	$1.2\times 10^{-1}$	2.1	$6.7\times 10^{-1}$	$2.5\times 10^{-2}$	1.7
3	$1.1\times 10^{0}$	$8.3\times 10^{-2}$	1.8	$5.0\times 10^{-1}$	$1.7\times 10^{-2}$	1.5
4	$8.6\times 10^{-1}$	$6.2\times 10^{-2}$	1.7	$4.0\times 10^{-1}$	$1.3\times 10^{-2}$	1.4
5	$7.1\times 10^{-1}$	$5.0\times 10^{-2}$	1.6	$3.4\times 10^{-1}$	$1.0\times 10^{-2}$	1.3
6	$6.1\times 10^{-1}$	$4.2\times 10^{-2}$	1.5	$2.9\times 10^{-1}$	$8.6\times 10^{-3}$	1.3
7	$5.3\times 10^{-1}$	$3.6\times 10^{-2}$	1.4	$2.6\times 10^{-1}$	$7.4\times 10^{-3}$	1.3
8	$4.7\times 10^{-1}$	$3.1\times 10^{-2}$	1.4	$2.3\times 10^{-1}$	$6.4\times 10^{-3}$	1.2
9	$4.3\times 10^{-1}$	$2.8\times 10^{-2}$	1.4	$2.1\times 10^{-1}$	$5.7\times 10^{-3}$	1.2
10	$3.9\times 10^{-1}$	$2.5\times 10^{-2}$	1.3	$1.9\times 10^{-1}$	$5.2\times 10^{-3}$	1.2
20	$2.1\times 10^{-1}$	$1.2\times 10^{-2}$	1.2	$1.0\times 10^{-1}$	$2.6\times 10^{-3}$	1.1
40	$1.1\times 10^{-1}$	$6.2\times 10^{-3}$	1.1	$5.3\times 10^{-2}$	$1.3\times 10^{-3}$	1.1
60	$7.2\times 10^{-2}$	$4.2\times 10^{-3}$	1.1	$3.6\times 10^{-2}$	$8.6\times 10^{-4}$	1.0
80	$5.5\times 10^{-2}$	$3.1\times 10^{-3}$	1.1	$2.7\times 10^{-2}$	$6.5\times 10^{-4}$	1.0
100	$4.4\times 10^{-2}$	$2.5\times 10^{-3}$	1.0	$2.2\times 10^{-2}$	$5.2\times 10^{-4}$	1.0

## Numerical verification

Having motivated the application of scaled saddle-point preconditioners to Stokes problems, we would like to illustrate numerically the effect of the scaling. In particular, it is important to observe the effectiveness of this strategy when state-of-the-art preconditioners are applied both exactly and inexactly (as an inexact application will generally result in a more efficient algorithm), and determine the potency of our approach for different finite element discretizations.

**Fig. 3:**
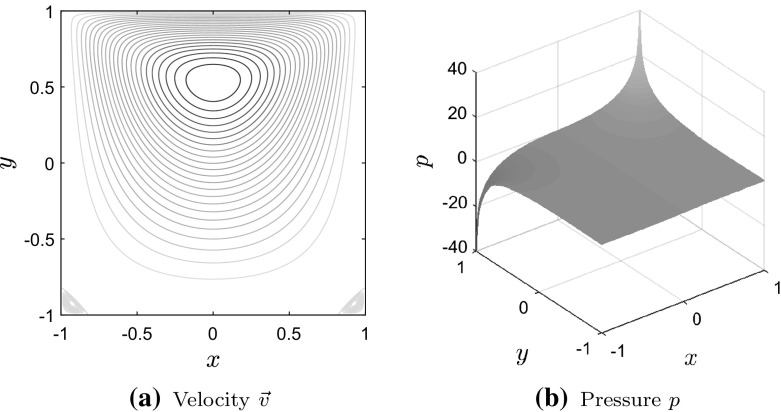
Solution plots of velocity $\vec {v}$ and pressure *p* for the regularized cavity problem

**Fig. 4:**
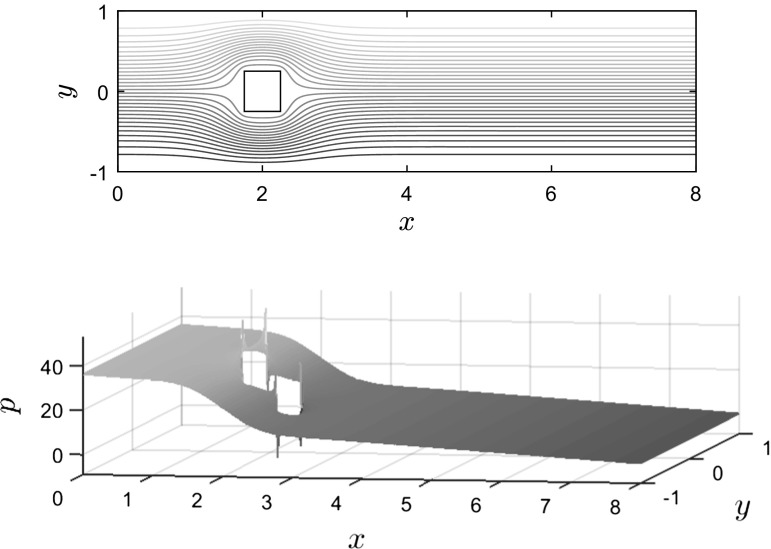
Solution plots of velocity $\vec {v}$ (*top*) and pressure *p* (*bottom*) for the obstacle flow problem

To ascertain this we run the preconditioned MINRES algorithm on particular test problems, to a preconditioned residual norm tolerance of $10^{-6}$, within the IFISS software system [9, 10, 22] in matlab. In particular, we examine the regularized cavity flow problem from Sect. 3, as well as an obstacle flow problem. The latter problem is posed on the channel $\Omega = [0,8]\times [-1,1]$ with the square obstacle $[\frac{7}{4},\frac{9}{4}]\times [-\frac{1}{4},\frac{1}{4}]$ removed (see Fig. 4). No-flow conditions are applied at the top and bottom walls, and at the obstacle boundary. We impose a Poiseuille flow condition, that is $v_{x}=1-y^{2}$, $v_{y}=0$, on the inflow boundary; we also specify a natural boundary condition on the outflow boundary. In Fig. 3 we present a streamline plot for the velocity solution of the cavity problem, and a plot of the pressure solution; for these plots we set $h=2^{-8}$ (corresponding to the finest mesh tested). In Fig. 4 we provide the same plots for the obstacle flow problem, with $h=2^{-7}$.

### The effect of the parameter $\alpha$

**Fig. 5:**
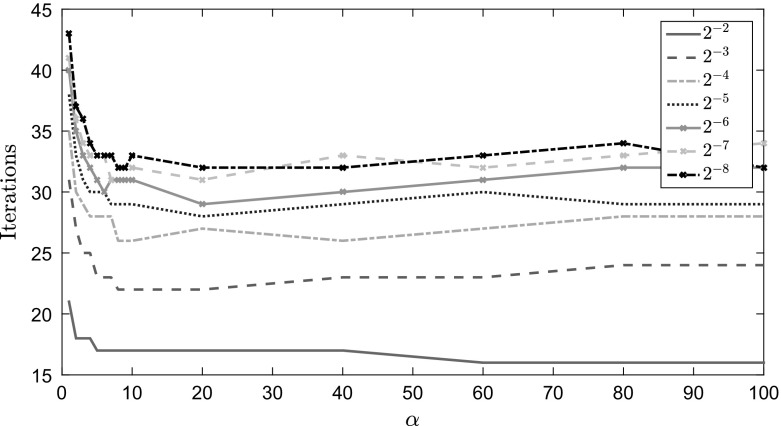
Representation of the effect of $\alpha$ on the MINRES iteration count for the cavity problem

In Table 8 we present iteration numbers for the MINRES solution of the regularized cavity problem using stabilized $Q_1 \textendash Q_1$ elements on a uniform mesh. Within the preconditioner, we use one AMG V-cycle with point damped Gauss-Seidel smoothing for the matrix ${\widehat{A}}$, and 10 steps of Chebyshev semi-iteration [12, 13, 30] for *H*. We present results for different values of the (uniform) mesh parameter *h*, as well as values of $\alpha$ within ${\mathcal {P}}_{\alpha }$. We observe that when $\alpha$ is increased, the iteration numbers clearly decrease, and there is hence a considerable benefit to applying the scaled preconditioner. This is observed for all values of mesh parameter tested. We present these results pictorially in Fig. 5, illustrating the effect of $\alpha$ for all values of *h* tested.

**Table 8:** Results for the cavity problem solved with $Q_1 \textendash Q_1$ finite elements, for a range of values of *h* and $\alpha$

$\alpha$	*h*						
	$2^{-2}$	$2^{-3}$	$2^{-4}$	$2^{-5}$	$2^{-6}$	$2^{-7}$	$2^{-8}$
1	21	31	35	38	40	41	43
2	18	27	30	33	35	36	37
3	18	25	29	31	33	34	36
4	18	25	28	30	32	33	34
5	17	23	28	30	31	33	33
6	17	23	28	30	30	33	33
7	17	23	28	29	31	31	33
8	17	22	26	29	31	32	32
9	17	22	26	29	31	32	32
10	17	22	26	29	31	32	33
20	17	22	27	28	29	31	32
40	17	23	26	29	30	33	32
60	16	23	27	30	31	32	33
80	16	24	28	29	32	33	34
100	16	24	28	29	32	34	32

### Effectiveness for different preconditioning options

We now wish to observe whether our approach is effective for a range of (exact and inexact) preconditioners, as well as for different finite element basis functions. In Table 10 we therefore present iteration numbers for the solution of the obstacle problem in such scenarios, with the different preconditioning strategies presented in Table 9. The matrix ${\widehat{A}}$ is either taken to be *A* or an AMG V-cycle applied to it; the preconditioner *H* for the Schur complement is either the diagonal of *Q* or 10 steps of Chebyshev semi-iteration applied to *Q*. We highlight that we also ran the same tests with $H=Q$, and obtained very similar results as when using Chebyshev semi-iteration. When $Q_1 \textendash P_0$ finite elements are used, *Q* is diagonal, so we only run the tests for preconditioner options 1 and 3. In all cases the mesh parameter is fixed as $h=2^{-7}$, and different values of $\alpha$ are again taken within ${\mathcal {P}}_{\alpha }$. We see that applying Chebyshev semi-iteration within the Schur complement approximation results in faster convergence than a diagonal approximation; using AMG to approximate the (1, 1)-block yields roughly similar convergence as an exact inverse for $Q_1 \textendash Q_1$ elements, but higher iteration counts for $Q_1 \textendash P_0$ and $Q_2 \textendash Q_1$ elements. Significantly, we once again observe the advantage of increasing $\alpha$ within the preconditioner—this behavior is replicated for all preconditioning options tested when stabilized finite elements are used. We highlight that each MINRES iteration requires the same computational operations for a given matrix system, and therefore a reduction in the iteration count results in a corresponding decrease in computing time. In the best case we observe a reduction of 30% in MINRES steps and hence CPU time, when increasing the value of $\alpha$ for a stabilized problem.

**Table 9:** Different preconditioner options

	Preconditioner
1	Full *A*, Diagonal of *Q*
2	Full *A*, Chebyshev semi-iteration for *Q*
$2^*$	Full *A*, Exact *Q*
3	AMG for *A*, Diagonal of *Q*
4	AMG for *A*, Chebyshev semi-iteration for *Q*

**Table 10:** Results for the obstacle flow problem with $h = 2^{-7}$ for different preconditioners, and for a range of $\alpha$ and element types

$\alpha$	$Q_1 \textendash Q_1$				$Q_1 \textendash P_0$		$Q_2 \textendash Q_1$			
	1	2	3	4	1	3	1	2	3	4
1	64	67	67	72	69	77	77	49	89	61
2	61	60	65	64	62	70	75	48	86	60
3	61	55	65	60	59	67	73	48	85	60
4	59	54	63	59	58	66	73	49	83	59
5	59	53	63	57	58	65	71	47	84	60
6	58	52	63	58	56	64	71	47	82	60
7	58	52	63	58	56	64	71	47	83	61
8	57	51	62	57	56	65	70	47	83	61
9	57	51	62	57	55	64	70	47	82	60
10	56	50	62	57	55	64	70	47	82	60
20	56	49	62	59	53	65	68	46	83	62
40	54	48	64	61	52	68	66	45	84	66
60	54	47	65	63	52	69	65	45	87	67
80	52	47	66	64	52	71	65	45	88	69
100	52	47	66	66	52	72	63	45	90	70

### Norm in which convergence is achieved

It is important to highlight that, although the classical stopping criteria for MINRES involves convergence of the relative preconditioned residual norm to a desired tolerance, we wish to achieve accurate solutions in measures that are not themselves influenced by the preconditioner. We have therefore calculated $\Vert {\varvec{r}}_k\Vert _2/\Vert {\varvec{r}}_0\Vert _2$ for the solutions obtained using our solver for all values of $\alpha$ tested. (Recall that our stopping criterion is $\Vert {\varvec{r}}_k\Vert _{{{\mathcal {P}}}_\alpha ^{-1}}/\Vert {\varvec{r}}_0\Vert _{{{\mathcal {P}}}_\alpha ^{-1}} < 10^{-6}$.) In Table 11 we state, for a range of basis functions and values of *h*, the ‘worst case’ relative residual norm achieved for the obstacle problem, and the value of $\alpha$ for which it was achieved. This verified that the solutions we obtained were accurate in a real sense, and not in a measure that was itself affected by the value of $\alpha$. In fact, for smaller values of *h* (i.e. problems of higher dimension), the accuracy of our solutions seemed to improve. We observed that the rate of convergence achieved was dictated by the factor $\mathcal {R}_{\alpha }$, as suggested by the analysis of Sect. 2.

**Table 11:** Worst case relative residual norm $\Vert {\varvec{r}}_k\Vert _2/\Vert {\varvec{r}}_0\Vert _2$, with corresponding values of $\alpha$ in parentheses, for the obstacle flow problem with different preconditioners, values of *h*, and element types

		$h=2^{-5}$	$h=2^{-6}$	$h=2^{-7}$
$Q_1-Q_1$	1	$1.3\times 10^{-7}$ (1)	$1.1\times 10^{-7}$ (2)	$6.5\times 10^{-8}$ (7)
	2	$1.0\times 10^{-7}$ (6)	$1.1\times 10^{-7}$ (6)	$9.2\times 10^{-8}$ (7)
	3	$1.2\times 10^{-7}$ (3)	$8.2\times 10^{-8}$ (1)	$6.4\times 10^{-8}$ (1)
	4	$1.4\times 10^{-7}$ (6)	$9.2\times 10^{-8}$ (3)	$4.8\times 10^{-8}$ (10)
$Q_1-P_0$	1	$1.3\times 10^{-7}$ (3)	$1.1\times 10^{-7}$ (2)	$7.4\times 10^{-8}$ (4)
	3	$1.2\times 10^{-7}$ (4)	$7.4\times 10^{-8}$ (1)	$4.5\times 10^{-8}$ (1)
$Q_2-Q_1$	1	$1.5\times 10^{-7}$ (40)	$8.2\times 10^{-8}$ (9)	$5.3\times 10^{-8}$ (6)
	2	$1.5\times 10^{-7}$ (40)	$8.6\times 10^{-8}$ (1)	$4.7\times 10^{-8}$ (1)
	3	$8.9\times 10^{-8}$ (80)	$6.9\times 10^{-8}$ (1)	$5.1\times 10^{-8}$ (1)
	4	$8.9\times 10^{-8}$ (80)	$6.4\times 10^{-8}$ (5)	$3.8\times 10^{-8}$ (40)

### General problems

**Fig. 6:**
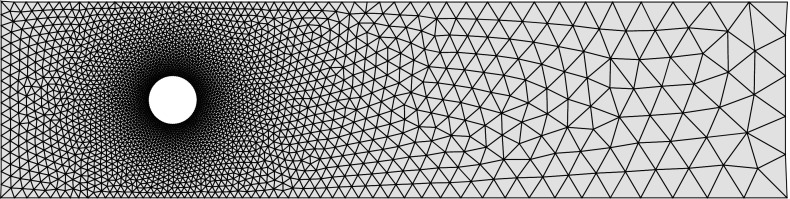
*G*0 mesh (22 348 degrees of freedom) for the circular obstacle problem

We now investigate whether our results hold for more general problems. In particular, we examine the solution of the three-dimensional cavity problem on $\Omega = [0,1]^3$, with $\vec {f} = \vec {0}$ and boundary conditions

$$\begin{aligned} \ v_{x}=1,~v_{y}=0,~v_{z}=0,\quad&\text {on }[-1,1]^2\times \{1\}, \\ \ v_{x}=v_{y}=v_{z}=0,\quad&\text {on }\partial \Omega \backslash \big ([-1,1]^2\times \{1\}\big ), \end{aligned}$$

where $\vec {v}=[v_{x},~v_{y},~v_{z}]^{T}$. As for the two-dimensional cavity problem, the flow is enclosed and so the preconditioned system is singular. We also computed a two-dimensional Stokes flow around a circular shaped obstacle using a highly unstructured mesh, *G*0, (see Fig. 6) and a uniformly refinement of it, mesh *G*1. These results were computed using the T-IFISS software package.^5^

Tables 12 and 13 show iteration numbers for different $\alpha$, with the preconditioners as in Table 9. For both problems we see qualitatively similar behavior to that in Table 10 for the 2D obstacle problem, so that increasing $\alpha$ reduces the number of iterations needed. This is mirrored by eigenvalue computations (not shown) which, in both cases, display qualitatively similar behavior to the 2D model problems.

**Table 12:** Results for the 3D cavity flow problem with $h = 2^{-3}$ and $P_2 \textendash P_1$ elements for different preconditioners, and for a range of $\alpha$

$\alpha$	1	2	3	4
1	55	49	58	53
10	49	43	52	48
100	43	35	53	47

**Table 13:** Results for the 2D circular obstacle flow problem for two meshes and $P_2 \textendash P_1$ elements for different preconditioners, and for a range of $\alpha$

$\alpha$	G0		G1	
	1	$2^*$	1	$2^*$
1	53	42	53	43
10	46	39	46	38
100	42	37	41	35

The *G*0 mesh has 22 348 degrees of freedom and the *G*1 mesh has 99 710 degrees of freedom

## Concluding remarks

This work shows that including a simple scaling to well-established block diagonal preconditioners for Stokes problems can result in significantly faster convergence when applying the preconditioned MINRES method. We demonstrated theoretically why this occurs by analyzing the eigenvalues of the preconditioned matrix ${{\mathcal {P}}}_\alpha ^{-1}{{\mathcal {A}}}$. In particular, the positive eigenvalues cluster near 1 as the scaling parameter is increased, with the negative eigenvalues also clustering and only approaching 0 slowly. We also show that the performance gains can be significant ($30\%$ reduction in CPU times) if a stabilized mixed approximation method is in use.

## Footnotes

1 We note that the condition $C=0$ is often required for a matrix to be defined as a saddle-point system. In this work we consider the more general definition, where *C* is required to be symmetric positive semi-definite.

2 The lower bounds on the positive and negative eigenvalues are shown in [1, Corollary 1], with the upper bounds on the positive and negative eigenvalues a result of [23, Lemma 2.2].

3 This follows from expressing the discrete inf-sup stability condition as a generalized eigenvalue problem, see [8, page 173].

4 Note that, even in exact arithmetic, matrices with tight clusters of eigenvalues do not in general give the same convergence curve as matrices with distinct eigenvalues located at the cluster centres, as discussed by Liesen and Strakoš for the Conjugate Gradient method [15, Sect. 5.6.5].

5
http://www.maths.manchester.ac.uk/djs/ifiss/tifiss.html.
